# Unmalted Rice, Wheat, and Sorghum Modulate Antioxidant Capacity, Nitrogen Metabolism, Alcohol Profile, and Sensory Quality of Craft Ale Beer

**DOI:** 10.3390/molecules31213935

**Published:** 2026-11-01

**Authors:** Cesar I. Mejia-Llontop, Carlos E. Tirado-Rodríguez, Manuel E. Ascate-Pasos, María de Fátima Arévalo-Oliva, Janet E. Muñoz-Agreda, Eudes Villanueva, Delia Rita Tapia-Blácido, Elza Aguirre

**Affiliations:** 1 Laboratorio de Microbiología y Toxicología de Productos Agroindustriales, Universidad Nacional del Santa (UNS), Urb. Av. Universitaria s/n, Nuevo Chimbote 02712, Ancash, Peru; 201023040@uns.edu.pe (C.I.M.-L.);; 2 Escuela de Posgrado, Universidad Nacional del Santa (UNS), Av. Universitaria s/n, Nuevo Chimbote 02712, Ancash, Peru; 3 Instituto de Investigación Tecnológica Agroindustrial, Universidad Nacional del Santa (UNS), Av. Universitaria s/n, Nuevo Chimbote 02712, Ancash, Peru; janet.munoz.agreda@gmail.com; 4 Departamento Académico de Ingeniería en Industrias Alimentarias, Universidad Nacional Autónoma de Tayacaja Daniel Hernández Morillo (UNAT), Jr. Bolognesi Nro. 418, Pampas 09156, Huancavelica, Peru; 5 Departamento de Química, Faculdade de Filosofia, Ciências e Letras de Ribeirão Preto, Universidade de São Paulo, Avenida do Café, S/N, Ribeirão Preto 14040-903, SP, Brazil

**Keywords:** wort, malt, multivariate analysis, fusel alcohols, L-serine

## Abstract

The use of unmalted cereal adjuncts represents a strategy to diversify craft beer and modify its functional properties. This study evaluated the effects of partially replacing barley malt with unmalted rice, wheat, and sorghum (20% and 40%) on the chemical composition, antioxidant capacity, free amino acid profile, alcohol profile, and sensory characteristics of Ale beer. Kolbach index (KI), international bitterness units (IBU), total phenolic content (TPC), antioxidant capacity (ORAC and ABTS), free amino acids, ethanol and higher alcohols, and sensory attributes were determined, and principal component analysis (PCA) was performed to explore the relationships among the evaluated variables. Cereal type, substitution level, and their interaction significantly affected most variables, indicating that the effect of increasing the substitution level depended on the cereal. All adjunct formulations produced less ethanol than the control (8.758%, *v v*^−1^), with the lowest value at 40% rice (4.561%); isoamyl alcohol was lower in all adjunct beers, whereas isobutanol did not differ significantly among formulations. The 40% sorghum formulation (T6) showed the highest TPC (316.66 μg gallic acid equivalents (GAE) 100 mL^−1^) and ORAC (oxygen radical absorbance capacity; 11.59 mmol trolox equivalents (TE) L^−1^), and, together with 20% sorghum (T5), the highest ABTS (2,2′-azinobis-(3-ethylbenzothiazoline-6-sulfonic acid)) values (up to 7.64 mmol TE L^−1^). In contrast, 40% rice markedly reduced TPC and antioxidant capacity, whereas the wheat formulations showed values similar to or lower than the control. The residual free amino acid profile (L-serine, arginine, cysteine, aspartic acid, threonine, and alanine, among others) also differed among formulations; because wort amino acids were not measured, these differences cannot be attributed specifically to amino acid release during mashing or to yeast assimilation. PCA explained 63.5% of the total variance and separated the beer formulations according to cereal type and substitution level. Among the formulations evaluated, 40% unmalted sorghum showed the highest antioxidant capacity while its overall acceptability did not differ from that of the control, although its ethanol content was lower than that of the control.

## 1. Introduction

Beer is an ancient alcoholic beverage that is widely consumed worldwide, with origins dating back more than 6000 years to the ancient Sumerians [1]. It is currently defined as the product obtained from the fermentation of wort derived primarily from malted barley and hops through the action of yeast under controlled conditions [2]. The global beer market remains highly significant, with increasing diversification driven by the growing demand for craft beers, which emphasize the use of natural ingredients and distinctive sensory profiles [3,4,5]. Craft beer is defined as beer produced by small, independent breweries and is characterized by innovation, the use of traditional or alternative ingredients, and the production of non-standardized beverages with sensory profiles that differ from those of industrial beers [5,6]. The bioactive potential of craft beer is primarily attributed to its phenolic compounds derived from hops and malt, which act as antioxidants capable of scavenging free radicals and have been reported to exhibit diverse biological activities, including photoprotective, antibacterial, anti-inflammatory, and anticancer effects [7]. The global craft beer market was valued at USD 161.49 billion in 2025 and is projected to grow from USD 184.10 billion in 2026 to USD 525.16 billion by 2034, at a compound annual growth rate (CAGR) of approximately 14% during the forecast period [8].

Despite the wide variety of ingredients used in brewing, malt, hops, and yeast remain the fundamental raw materials. Depending on the yeast strain employed, beers are primarily classified as Ale, fermented by *Saccharomyces cerevisiae*, and Lager, fermented by *Saccharomyces pastorianus*, each exhibiting distinct fermentation conditions and sensory characteristics [2,9]. Barley malt plays a central role in beer production because it provides fermentable sugars, nitrogenous compounds, and enzymes required for mashing and fermentation, thereby influencing the color, flavor, and bioactive properties of the final product [10,11,12]. However, due to economic and technological factors, the use of unmalted adjunct grains such as rice, wheat, and sorghum has become increasingly common [13,14]. Although the incorporation of unmalted adjuncts has been widely adopted in the brewing industry, their use remains a subject of debate, particularly in craft brewing [15]. From a technological perspective, the partial replacement of barley malt with unmalted grains may significantly affect enzymatic activity during mashing, reducing saccharification efficiency and modifying wort composition [13]. Consequently, variations may occur in key brewing parameters such as wort density, diastatic power, kolbach index (KI), alcohol content, and fermentable sugar profile [16,17]. Furthermore, the incorporation of unmalted adjuncts influences the chemical composition of beer, particularly its phenolic compounds and other bioactive metabolites, which are closely associated with antioxidant capacity [7,18]. These compounds play an important role in oxidative stability and may contribute to the potential health benefits of beer [7]. Nevertheless, their concentration depends on both the type of adjunct and the substitution level employed [13]. From a sensory perspective, the use of unmalted grains may alter attributes such as color, aroma, flavor, and body, thereby influencing consumer acceptance [19]. Accordingly, sensory analysis represents an essential tool for evaluating the quality of the final product and its market potential [20]. For example, rice is widely used as a brewing adjunct because of its high carbohydrate content and its ability to produce lighter and more stable beers, although its low enzymatic activity and susceptibility to oxidation may present technological challenges [13,21]. In contrast, wheat contributes higher protein content, improves fermentation performance, and provides distinctive sensory characteristics, whereas sorghum has gained increasing attention as a gluten-free alternative despite its relatively low diastatic power and associated technological limitations [13,14,22].

Previous research has investigated starchy and unmalted cereal adjuncts in brewing, although the objectives and experimental approaches have varied across studies. Cadenas et al. [13] reviewed the influence of starchy adjuncts on the sensory and nutritional properties of beer, whereas Schnitzenbaumer and Arendt [23] reviewed brewing applications involving up to 40% unmalted oats and sorghum. Yorke et al. [24] experimentally evaluated the effects of unmalted barley, wheat, rice, and maize on the analytical and sensory characteristics of Lager beer, demonstrating that the effects depended on the cereal type and inclusion level. Cela et al. [25] investigated the brewing suitability of unmalted cereals and pseudocereals for gluten-free beer production, considering their technological performance and the physicochemical and sensory characteristics of the resulting beers. In contrast, Baillière et al. [26] compared ten unmalted alternative cereals and pseudocereals based on raw-material characteristics relevant to the brewing process. Collectively, these studies provide important insights into the technological suitability of alternative cereals and their effects on beer quality. However, they differ from the present study in their cereal combinations, experimental objectives, brewing conditions, or analytical scope.

Ale beers constitute an appropriate model system for investigating these effects because of their technological versatility and complex sensory characteristics [2]. In addition, Ale fermentation conditions promote the formation of volatile compounds that directly influence the aromatic profile of the beer [27].

The novelty of the present study lies in the integration of a comparative experimental design with a multidimensional characterization of craft Ale beer produced using three unmalted cereal adjuncts at two substitution levels. By evaluating these formulations under comparable brewing conditions, the study examines how cereal type and substitution level are associated with differences in physicochemical properties, antioxidant capacity, nitrogen-related parameters, free amino acid profiles, alcoholic composition, and sensory characteristics. Multivariate analysis is additionally employed to explore relationships among these variables and identify the parameters that contribute to the differentiation of beer formulations. This integrated framework provides new comparative evidence on how the use of different unmalted cereals and their incorporation levels may shape multiple dimensions of craft Ale beer quality.

Accordingly, this study aimed to evaluate the effects of partially replacing barley malt with unmalted rice, wheat, and sorghum at two substitution levels (20% and 40%) on the physicochemical properties, antioxidant capacity, nitrogen-related parameters, free amino acid profiles, alcoholic composition, and sensory characteristics of craft Ale beer. Multivariate analysis was also used to explore relationships among the measured variables and distinguish beer formulations according to adjunct type and substitution level. By integrating these complementary assessments under comparable brewing conditions, this study provides a comparative evaluation of the effects of these three unmalted cereal adjuncts on craft Ale beer quality.

## 2. Results and Discussion

### 2.1. Physicochemical Composition of Raw Materials

The proximate composition of the raw materials (Table 1) revealed statistically significant differences (*p* < 0.05) in all the components evaluated, including protein, carbohydrate, and total dietary fiber (TDF) contents, which are associated with technological performance during the mashing process. Barley malt exhibited the highest carbohydrate content (83.220%), consistent with its role as the primary source of fermentable extract in brewing owing to its high starch content and the amylolytic enzymes developed during malting [28,29]. However, carbohydrates were calculated by difference and therefore represent total carbohydrates, including dietary fiber, rather than fermentable sugars, and enzymatic activity was not measured. In contrast, sorghum presented the highest protein (11.126%) and TDF (30.431%) contents among the evaluated cereals. These compositional characteristics may negatively affect wort filtration and extract recovery during brewing [30]. According to Villicaña and Saldivar [31], non-starch polysaccharides and condensed tannins in sorghum can increase wort viscosity and limit the hydrolytic activity of α-amylase and β-amylase during saccharification, and sorghum has been described as a cereal rich in structural polysaccharides and phenolic compounds but with relatively low diastatic power [32]; these effects were not measured in the present study. Rice exhibited the lowest protein concentration (8.362%), consistent with previous studies describing rice as a low-nitrogen adjunct commonly used to produce lighter beers [17,23], which may result in a lower nitrogen supply to the wort. The protein content of wheat (10.245%) was higher than that of barley malt (9.955%) and lower than that of sorghum. These compositional differences suggest that each adjunct may influence brewing performance differently; their actual effects on wort composition, fermentation, and beer quality are addressed in the following sections on the finished beers.

### 2.2. Volatile Alcohol Composition of Beers

The alcohol profile (Table 2) showed that the partial substitution of barley malt with unmalted cereal adjuncts significantly modified the ethanol and isoamyl alcohol concentrations of the beers. These variations may be related to changes in wort composition, particularly fermentable sugar availability, free amino nitrogen (FAN), and amino acid precursors, which regulate yeast metabolism during alcoholic fermentation [28]; however, wort composition and fermentation kinetics were not measured in this study. All beer formulations containing unmalted adjuncts produced significantly lower ethanol concentrations than the control beer brewed exclusively with barley malt (8.758%; *p* < 0.05). Among all treatments, T2 (40% rice + 60% barley malt) exhibited the lowest ethanol concentration (4.561%), representing an approximately 47.9% reduction compared with the control. The decreased ethanol production in the rice formulations may be associated with a reduced availability of fermentable sugars generated during mashing, since increasing the proportion of unmalted cereals dilutes the diastatic potential of the base malt and reduces the activities of α-amylase and β-amylase responsible for starch hydrolysis [33,34,35]; however, impaired yeast performance related to limitations in assimilable nitrogen or other wort characteristics cannot be excluded. The marked difference between T1 (20% rice; 6.473%) and T2 (40% rice) further indicates that increasing the proportion of unmalted rice progressively reduced the ethanol concentration. Similar observations were reported by Ceccaroni et al. [36], who observed reduced extract recovery and lower alcohol contents in rice-based beers, and by Cadenas et al. [13], who found that increasing the proportion of unmalted rice and maize decreased fermentable extract availability and final ethanol concentration in craft beers. Wheat formulations (T3 and T4) produced higher ethanol concentrations (8.112% and 7.230%) than the rice and sorghum formulations, although both remained significantly lower than the control. This may be related to the protein content of wheat (Table 1) and its soluble nitrogenous compounds [37], and to the easier gelatinization and hydrolysis of wheat starch compared with sorghum starch [24], but these mechanisms were not evaluated here.

The sorghum formulations (T5, 6.805%, and T6, 6.418%) showed intermediate ethanol concentrations, lower than those of the control and the wheat formulations but higher than that of T2; T6 did not differ significantly from T1. Although sorghum contains considerable amounts of starch, its endosperm proteins are dominated by kafirins, hydrophobic storage proteins that restrict enzyme accessibility during mashing [38] and form dense cross-linked structures around starch granules that limit gelatinization and enzymatic hydrolysis [39]. Moreover, sorghum contains condensed tannins and other phenolic compounds capable of partially inhibiting hydrolytic enzymes [31]. These characteristics may account for the lower ethanol concentrations observed in the sorghum beers compared with the all-malt control, although starch conversion and fermentable sugars were not measured.

From a technological perspective, the lower ethanol concentration observed in T2 suggests that high levels of unmalted rice substitution may require process optimization to maintain adequate fermentation. This could be achieved by adjusting mashing conditions to improve starch hydrolysis and fermentable sugar production, together with appropriate management of yeast pitching rate, assimilable nitrogen, and fermentation conditions. The use of exogenous hydrolytic enzymes could also be considered when high proportions of unmalted rice are used. However, these strategies were not evaluated in the present study and should be experimentally validated in future research.

Regarding higher alcohols, only isoamyl alcohol differed significantly among the seven formulations (*p* < 0.05): all adjunct beers (0.014–0.016%) had significantly lower concentrations than the control (0.022%), with the lowest value in T2 (0.014%). Isobutanol did not differ significantly among the formulations (0.182–0.187%; Table 2), although the factorial analysis of T1–T6 detected a very small effect of cereal type on isobutanol (0.182–0.185%) and significant cereal × level interactions for both isobutanol and isoamyl alcohol (*p* ≈ 0.04; Table 6). *n*-Propanol was not quantified in this study. Higher alcohols are mainly synthesized through the Ehrlich pathway, in which branched-chain amino acids such as valine, leucine, and isoleucine undergo transamination, decarboxylation, and reduction to produce fusel alcohols [40]; isobutanol derives mainly from valine and isoamyl alcohol from leucine. Although present at relatively low concentrations, these volatile compounds contribute to the aroma of Ale beers depending on their concentration.

The relationship between nitrogen availability and higher alcohol formation was not straightforward in the present study. Isoamyl alcohol concentrations were significantly lower in all adjunct beers (0.014–0.016%) than in the control (0.022%), whereas isobutanol remained statistically unchanged (Table 2). This compound-specific response is consistent with the complexity of yeast amino acid metabolism, as higher alcohol formation depends not only on nitrogen availability but also on amino acid uptake, precursor metabolism, and yeast physiological regulation [28,40,41,42]. Although nitrogen limitation has been reported to modify amino acid catabolism and, under certain conditions, increase fusel alcohol production [28,41,42], such an increase was not observed in the present study. The Kolbach index varied among formulations (Table 3), but it represents the relationship between soluble and total nitrogen and should not be interpreted as a direct measurement of yeast-assimilable nitrogen (YAN). Furthermore, the amino acid concentrations reported in Table 4 represent residual levels in the finished beers rather than the initial availability of amino acids in the wort. Although valine and isoleucine were detected only in the control, and leucine was detected in the control and T4, these findings cannot establish the extent to which these amino acids were available to yeast during fermentation or converted into their corresponding higher alcohols. Overall, the results indicate that partial malt substitution altered isoamyl alcohol concentrations without producing a comparable change in isobutanol. However, because YAN, wort amino acid concentrations, and fermentation kinetics were not measured, the observed differences cannot be attributed specifically to nitrogen limitation. Direct measurements of YAN and amino acid consumption during fermentation would be necessary to clarify the mechanisms underlying these responses.

From a technological perspective, the present results show that both the type of adjunct cereal and the substitution level influenced the ethanol concentration of the beers (significant cereal × level interaction; Table 6). Wheat-based formulations showed the highest ethanol concentrations among the adjunct beers, whereas the 40% unmalted rice formulation (T2) showed the greatest reduction, indicating that high rice substitution may compromise ethanol production under the conditions evaluated. Therefore, the use of high proportions of unmalted rice should be accompanied by optimization of the mashing and fermentation conditions. Sorghum also reduced ethanol production relative to the control without increasing the concentration of higher alcohols.

In summary, partial substitution of barley malt with unmalted cereals consistently lowered the ethanol and isoamyl alcohol concentrations relative to the all-malt control, with magnitudes that depended on the cereal and the substitution level, whereas isobutanol remained essentially unchanged. The mechanisms underlying these differences (fermentable sugar availability, assimilable nitrogen, and the structural characteristics of each cereal) were not directly evaluated and should be addressed in future work.

### 2.3. Kolbach Index and Bitterness

The KI values showed statistically significant differences among the formulations (*p* < 0.05), with the highest values observed in T4 and the control and the lowest in T6 and T2 (Table 3). The KI is conventionally used to describe the relationship between soluble and total nitrogen and, in malt-based systems, is commonly associated with the degree of protein modification [34]. However, when unmalted cereals are incorporated into the grist, its interpretation becomes less straightforward because the nitrogen contribution and solubilization behavior of the different cereal components may vary. Therefore, in the present study, differences in KI are interpreted primarily as relative differences in nitrogen solubilization during mashing rather than as direct indicators of proteolytic modification or amino acid availability to yeast. The KI decreased when rice increased from 20% (T1) to 40% (T2) and, numerically, when sorghum increased from 20% (T5) to 40% (T6), although the latter difference was not significant, whereas it increased with wheat inclusion (T3 to T4). This indicates that the effect of the cereal adjuncts depended on both cereal type and incorporation level. The numerically lowest KI, observed in T6 (not significantly different from T2), may be associated with differences in the nitrogen composition and solubilization behavior of sorghum proteins. Kafirins are major storage proteins in sorghum and have been described as relatively hydrophobic and resistant to enzymatic hydrolysis [38], and differences in the composition and structure of the sorghum matrix may also affect nitrogen solubilization during mashing [43,44]. However, protein hydrolysis and individual protein fractions were not assessed; therefore, these interpretations should be considered possible explanations rather than demonstrated mechanisms. T1 and T4 showed KI values statistically comparable to those of the control, whereas T2 and T6 showed lower values. These differences were accompanied by variations in the residual amino acid profile and in ethanol concentration, although the present study does not establish a causal relationship between KI and these parameters.

Regarding bitterness, expressed as international bitterness units (IBU) determined spectrophotometrically (Table 3), significant differences were observed among the formulations (*p* < 0.05). Formulation T5 (20% sorghum + 80% malt) exhibited the highest IBU value, whereas T3 showed the lowest value. These results indicate that both the type of cereal and the incorporation level influenced the spectrophotometric bitterness response of the beers.

Nevertheless, the differences among the formulations should be interpreted with caution. The ASBC Beer-23A method is based on the extraction of acidified beer with isooctane, followed by absorbance measurement at 275 nm [45]. Consequently, the spectrophotometric response may be attributed to hop-derived iso-α-acids as well as other compounds extracted into the organic phase that absorb in the UV region, including α-acids, polyphenols, and humulinones [46]. Therefore, the relatively high IBU value observed for T5 should not be interpreted as direct evidence of a proportionally higher concentration of iso-α-acids.

Sorghum contains phenolic acids, flavonoids, and other phenolic compounds [47], which could contribute to the pool of UV-absorbing compounds present in the beer matrix. However, the analytical method employed in the present study does not allow the individual contribution of these compounds to be distinguished from that of hop-derived bitter compounds. Furthermore, the contrasting values observed between T5 (20% sorghum) and T6 (40% sorghum) suggest that the measured IBU response was not directly related to the proportion of sorghum incorporated.

Although the results demonstrate that both the type of cereal and the incorporation level affected the spectrophotometrically determined bitterness of the beers, these findings should be corroborated by additional chromatographic analyses of individual iso-α-acids to determine whether the differences in IBU values correspond to proportional differences in their actual concentrations.

### 2.4. Free Amino Acid Content

The results presented in Table 4 demonstrated that the partial replacement of barley malt with unmalted rice, wheat, and sorghum significantly (*p* < 0.05) modified the free amino acid composition of Ale beers, particularly affecting the concentrations of L-serine, arginine, L-cysteine, aspartic acid, L-threonine, and alanine. Free amino acids constitute one of the most important indicators of nitrogen metabolism throughout brewing because they reflect both the extent of proteolytic degradation during mashing and the assimilation of FAN by *S. cerevisiae* during alcoholic fermentation [48]. Consequently, variations in amino acid profiles provide valuable information regarding yeast nutritional status, fermentation performance, aroma formation, and the final quality of beer.

The residual free amino acid profile of the finished beers (Table 4) integrates the amino acids released during mashing and those consumed by yeast during fermentation; since amino acids were not determined in the wort, these two contributions cannot be separated in the present study. Differences among cereals may be related to protein composition, proteolytic susceptibility, and nitrogen availability, which can in turn influence yeast metabolism and the formation of volatile compounds through the Ehrlich pathway [40]. L-Serine was the predominant amino acid in all beer formulations, with the highest concentration in T6 (346.04 µg mL^−1^), followed by T5 (344.00 µg mL^−1^) and T1 (342.80 µg mL^−1^), which did not differ from each other. Thus, serine was higher than in the control (323.73 µg mL^−1^) in all rice and sorghum formulations. Serine is an important metabolite in yeast physiology, contributing to phospholipid and nucleotide synthesis and central carbon metabolism, and it serves as a precursor for glycine and cysteine biosynthesis [40,49]. Residual serine could reflect a lower uptake by yeast or a higher release during mashing; the data do not discriminate between these possibilities. Less attenuated fermentations may retain higher concentrations of residual amino acids [28], but the pattern observed here does not follow the ethanol results consistently: T2, which had the lowest ethanol concentration, had lower serine than T1, T5, and T6, and T1 and T6 had similar ethanol concentrations (Table 2).

Conversely, serine concentrations in the wheat formulations (T3, 320.53 µg mL^−1^; T4, 312.86 µg mL^−1^) were significantly lower than in the control, with T4 showing the lowest value; this may indicate a greater uptake during fermentation or a lower release during mashing. Wheat proteins have been reported to be more susceptible to enzymatic degradation than sorghum kafirins [37], and worts with elevated FAN concentrations stimulate rapid amino acid uptake during the early stages of fermentation [50], but FAN and wort amino acids were not measured here. Arginine was detected at relatively constant concentrations (168.61–169.32 µg mL^−1^) in the control, T1, T3, T4, and T6, but it was not detected in T2 and T5. Arginine is one of the principal nitrogen sources available to brewing yeast and one of the most abundant amino acids in wort [48]; it is metabolized through the arginase pathway, releasing urea and subsequently ammonium [49]. Therefore, residual arginine reflects the balance between its release during mashing and its assimilation by yeast.

The absence of arginine in T2 and T5 may reflect a greater yeast uptake or a lower release during mashing; these alternatives cannot be distinguished with the present data. L-Cysteine was detected only in the control beer (244.86 µg mL^−1^) and T1 (247.97 µg mL^−1^), and it was not detected in any other formulation. Cysteine is involved in cellular redox regulation and is the principal precursor for glutathione synthesis in yeast [51], and sulfur-containing amino acids can contribute to the formation of volatile sulfur compounds [42]. The absence of cysteine in most adjunct formulations may be associated with its utilization during fermentation or with a lower release from the cereal proteins, but neither possibility was evaluated. Aspartic acid was higher in T3, T4, T5, and T6 (106.43–114.19 µg mL^−1^) than in the control (69.69 µg mL^−1^), had an intermediate value in T1 (104.36 µg mL^−1^), and was not detected in T2. Aspartic acid occupies a central position in nitrogen metabolism because it serves as a precursor for the biosynthesis of lysine, methionine, threonine, and other amino acids [49].

The higher residual aspartic acid concentrations in most adjunct beers may reflect either enhanced protein degradation during mashing or reduced assimilation by yeast, whereas its absence in T2 shows that this response was not common to all adjunct formulations. L-Threonine was higher in all adjunct beers (250.05–264.45 µg mL^−1^) than in the control (243.57 µg mL^−1^), with the highest concentration in T5 (264.45 µg mL^−1^), followed by T2 (260.21 µg mL^−1^) and T6 (258.70 µg mL^−1^). This pattern does not follow the protein content of the cereals (Table 1), since T2, containing the cereal with the lowest protein content, showed the second highest value. Threonine is a precursor of *n*-propanol through the Ehrlich pathway [40]; however, *n*-propanol was not quantified in this study, and therefore no relationship between threonine and higher alcohols can be established.

Alanine concentrations varied little among treatments (230.27–237.27 µg mL^−1^): T5 and T2 showed the highest values, T4 and T6 the lowest, and none of the adjunct beers differed significantly from the control. Alanine is synthesized primarily through pyruvate transamination and links glycolysis with amino acid metabolism [49]; given the small magnitude of the differences, no metabolic interpretation is proposed.

Valine and isoleucine were detected only in the control beer (34.31 and 80.86 µg mL^−1^, respectively), and leucine only in the control (40.61 µg mL^−1^) and T4 (40.44 µg mL^−1^). Branched-chain amino acids are precursors of isobutanol (valine) and isoamyl alcohol (leucine) through the Ehrlich pathway [40]. However, their absence in most adjunct beers was not accompanied by higher fusel alcohol concentrations: isobutanol did not differ among formulations and isoamyl alcohol was lower in all adjunct beers than in the control (Table 2). Therefore, the present data do not allow the absence of these amino acids to be interpreted as an increased catabolism through the Ehrlich pathway; it may also reflect a lower release during mashing or concentrations below the detection limit, which cannot be distinguished without wort data. Notably, the control beer, the only one with detectable valine and isoleucine, also had the highest isoamyl alcohol concentration.

Tyrosine was detected at very similar concentrations (26.61–26.76 µg mL^−1^) in T1, T2, T3, T4, and T6, and was not detected in the control or T5; given the similarity of these values, the differences should be interpreted with caution. Glycine (74.21 µg mL^−1^) and proline (97.23 µg mL^−1^) were detected exclusively in T3. Proline is one of the few amino acids poorly assimilated by *S. cerevisiae* under anaerobic brewing conditions [48], so its presence only in T3 may be related to differences in proteolysis or amino acid utilization in this formulation, although this was not evaluated.

Collectively, these results show that the partial substitution of barley malt modified the residual free amino acid profile of the beers in a cereal- and level-dependent manner. Serine was higher than in the control in the rice and sorghum formulations (highest in T6), threonine was higher in all adjunct formulations (highest in T5), the wheat formulations had the lowest serine concentrations and high aspartic acid concentrations, and glycine and proline were detected only in T3. Because amino acids were measured only in the finished beers, these differences cannot be attributed to a specific change in amino acid release during mashing or in nitrogen assimilation during fermentation, and they should not be interpreted as differences in nitrogen utilization or fermentation efficiency. The determination of FAN and amino acids in the wort would be required to clarify these mechanisms [28].

### 2.5. Total Phenolic Content and Antioxidant Capacity

The results presented in Table 5 showed that the partial substitution of barley malt with unmalted cereals significantly (*p* < 0.05) affected the TPC as well as the antioxidant capacity measured by both the oxygen radical absorbance capacity (ORAC) and ABTS radical scavenging assays. T6 (40% sorghum) showed the highest values for all three parameters, whereas T5 (20% sorghum) had the second highest TPC and an ABTS value that was not significantly different from that of T6, but its ORAC did not differ significantly from that of the control. Among the rice formulations, only T2 (40% rice) showed the lowest values for all three parameters; T1 (20% rice) had a higher TPC than the control, a lower ORAC, and an ABTS value similar to that of the control. These findings suggest that the botanical origin of the adjunct and its level of incorporation contributed to differences in the phenolic and antioxidant profiles of the finished beers, although matrix-dependent effects and contributions from hop- and malt-derived compounds cannot be excluded.

Significant differences in TPC were observed among the beer formulations. The highest TPC values were recorded in T6 (316.66 μg GAE 100 mL^−1^) and T5 (277.89 μg GAE 100 mL^−1^), both higher than the control (230.52 μg GAE 100 mL^−1^), while T2 exhibited the lowest concentration (135.98 μg GAE 100 mL^−1^). T1 (260.18 μg GAE 100 mL^−1^) also exceeded the control, whereas T3 did not differ from it and T4 (189.83 μg GAE 100 mL^−1^) was lower. The higher TPC of the sorghum beers may be partly associated with the phenolic composition of sorghum, although contributions from hop-derived compounds and matrix-dependent effects during brewing cannot be excluded. Sorghum phenolics are natural antioxidants capable of donating hydrogen atoms, chelating transition metals, and scavenging reactive oxygen species [47], and sorghum accumulates substantial amounts of phenolic compounds within the pericarp and testa, making it one of the richest cereal sources of dietary antioxidants. The behavior of sorghum polyphenols during malting has also been examined [52]. The higher TPC of T1 compared with the control was not expected from the low phenolic content generally attributed to rice and is not explained by the present data. Importantly, the Folin–Ciocalteu assay is not specific to phenolic compounds; it measures the reducing capacity of the sample and may also respond to non-phenolic reducing substances [53]. Consequently, the higher TPC measured in T1 does not necessarily indicate a higher concentration of phenolic compounds. In contrast, ORAC primarily evaluates the capacity of antioxidants to inhibit peroxyl-radical-induced oxidation through hydrogen atom transfer [54]. Therefore, constituents contributing to the Folin–Ciocalteu response may not contribute to ORAC activity to the same extent. Matrix-dependent effects and differences in the chemical reactivity of beer constituents may also have contributed to the observed discrepancy. However, because individual phenolic compounds and non-phenolic reducing substances were not identified or quantified in this study, their specific contributions cannot be established.

The higher TPC of the sorghum beers is in line with reports that phenolic-rich ingredients increase the phenolic content of specialty beers [55] and that phenolic compounds contribute to the overall antioxidant properties of malt and beer [56].

The antioxidant capacity measured using the ORAC assay was highest in T6 (11.594 mmol TE L^−1^), followed by T5 (10.216 mmol TE L^−1^) and the control (9.867 mmol TE L^−1^), which did not differ significantly from each other, whereas T2 showed the lowest value (3.383 mmol TE L^−1^). The ORAC values of T1 (6.903 mmol TE L^−1^), T3 (6.543 mmol TE L^−1^), and T4 (5.056 mmol TE L^−1^) were significantly lower than that of the control. The ORAC assay evaluates antioxidant activity through hydrogen atom transfer reactions by measuring the capacity of antioxidants to neutralize peroxyl radicals, one of the major reactive oxygen species involved in oxidative deterioration [57]. ORAC therefore followed the TPC trend at the extremes (T6 highest and T2 lowest), but not in all formulations: T1 had a higher TPC than the control but a lower ORAC.

Among the adjunct beers, the ORAC values of the sorghum formulations, particularly T6, are consistent with a contribution of phenolic compounds from sorghum to radical scavenging activity, since these compounds can inhibit oxidative chain reactions by donating hydrogen atoms and stabilizing free radicals [58]. The low ORAC value of T2 may be related to the low phenolic content generally attributed to rice [17] and to the lower proportion of malt in the grist (60%); however, T1, with the same cereal at 20%, did not show a lower TPC than the control. Thus, the reduction in antioxidant capacity was observed mainly at the 40% rice level and cannot be generalized to all rice formulations. A relationship between cereal phenolics and ORAC values has previously been reported: Adom and Liu [59] described phenolic compounds as principal contributors to the antioxidant capacity of whole grains, and Zhao et al. [60] reported positive correlations between total phenolic content and antioxidant activity in commercial beers.

The antioxidant activity evaluated using the ABTS assay also differed significantly among the beer formulations. The highest values were obtained for T5 (7.638 mmol TE L^−1^) and T6 (7.302 mmol TE L^−1^), which did not differ from each other and were higher than that of the control (6.806 mmol TE L^−1^), whereas T2 exhibited the lowest value (3.635 mmol TE L^−1^). Unlike ORAC, which is based primarily on hydrogen atom transfer, the ABTS assay measures antioxidant activity mainly through electron transfer mechanisms by quantifying the ability of antioxidants to reduce the ABTS^•+^ radical cation [61].

The higher ABTS activity of the sorghum beers is consistent with the high content of condensed tannins, flavonoids, and phenolic acids characteristic of this cereal; Awika et al. [62] reported a high antioxidant activity of sorghum extracts, attributed to the interaction among different classes of phenolic compounds. In contrast, T2 showed a significantly lower ABTS activity than the control, whereas T1 did not differ from the control (6.647 vs. 6.806 mmol TE L^−1^). Thus, the low antioxidant capacity associated with rice in the literature [17] was observed in this study only at the 40% inclusion level.

The wheat formulations (T3 and T4) showed a TPC similar to (T3) or lower than (T4) that of the control, and ORAC and ABTS values lower than those of the control but intermediate between the sorghum formulations and T2 (T3, 6.543 and 5.235; T4, 5.056 and 4.842 mmol TE L^−1^, respectively). Wheat contains ferulic acid and related hydroxycinnamic acids, but most of these compounds are bound to arabinoxylans and other cell wall components, which may limit their extraction during mashing [63]; this was not evaluated here. The three assays agreed at the extremes (T6 highest and T2 lowest), indicating that phenolic compounds are likely to contribute to the antioxidant capacity of the beers; however, the agreement was not complete (e.g., T1 and T3), and no correlation analysis between TPC and the antioxidant assays was performed. Therefore, phenolic compounds cannot be considered the sole contributors to the antioxidant capacity, and other compounds present in the beer matrix may also contribute.

Numerous studies have reported similar relationships between phenolic composition and antioxidant activity. Nardini and Foddai [55] demonstrated that the antioxidant properties of specialty beers depend primarily on both the concentration and composition of phenolic compounds present in the beverage. Likewise, Gorjanović et al. [64] observed significant positive correlations between total polyphenol content and several antioxidant assays, including spectrophotometric and electrochemical methods. Similar conclusions were reached by Zhao et al. [60], who reported strong correlations between phenolic profiles and antioxidant capacity in commercial beers evaluated using both ORAC and ABTS based methodologies.

Beer polyphenols have been associated with technological roles such as delaying oxidation and improving flavor stability; however, oxidative stability and shelf life were not evaluated in this study, so no conclusions can be drawn on them. Collectively, the results show that the effect of the cereal adjunct on the antioxidant profile of Ale beer depended on the cereal and the substitution level. Among the evaluated formulations, 40% sorghum (T6) showed the highest TPC, ORAC, and ABTS values, and 20% sorghum (T5) showed higher TPC and ABTS than the control, whereas 40% rice (T2) markedly reduced all three parameters. These results refer to the in vitro antioxidant capacity measured in the beers and do not by themselves demonstrate functional or health benefits. In this respect, T6 appears as the most promising formulation in terms of antioxidant capacity, whereas the lower acceptability of T5 and the lower ethanol content of both sorghum formulations (Table 2) should also be considered.

### 2.6. Effects of Cereal Type and Inclusion Level on Beer Characteristics

The factorial ANOVA (T1–T6) showed that beer characteristics were significantly influenced by the interaction between cereal type and inclusion level (Table 6). Significant interactions were observed for the KI, bitterness, ethanol, TPC, ORAC, ABTS, all the amino acids evaluated and, with lower significance, isoamyl alcohol (*p* = 0.0363) and isobutanol (*p* = 0.0414), indicating that the effect of increasing inclusion from 20% to 40% depended on the cereal used. This behavior is consistent with studies reporting differential responses when rice and sorghum are used as alternative cereals in brewing [65,66,67].

The KI and amino acid profile revealed differences in nitrogen-related characteristics. Increasing wheat inclusion increased KI (T3 to T4), whereas rice (T1 to T2) and sorghum (T5 to T6) showed the opposite trend; consequently, the main effect of inclusion level on KI was not significant (*p* = 0.0617). Likewise, the differences observed in the amino acids (*p* < 0.05) indicate that the residual amino acid profile depended on the specific cereal × inclusion combination, possibly due to differences in protein composition and enzymatic activity during mashing [67]. Ethanol also showed a significant interaction, with higher concentrations in wheat-containing beers and a marked reduction in the 40% rice treatment. In contrast, higher alcohol concentrations were less sensitive to the treatments: cereal type and the interaction were significant for isobutanol, and only the interaction for isoamyl alcohol, with differences of very small magnitude (0.182–0.185% and 0.014–0.016%, respectively).

Bitterness also showed a cereal-dependent response, increasing with wheat and rice inclusion but decreasing markedly with sorghum. This response may be associated with differences in wort composition and in the compounds that absorb in the UV region and contribute to the spectrophotometric IBU value (see Section 2.3). The most consistent effects were observed for antioxidant properties. Increasing wheat and rice inclusion reduced TPC and ORAC, whereas sorghum increased both parameters; ABTS decreased with rice inclusion and did not change significantly with wheat or sorghum inclusion. This behavior agrees with studies highlighting the contribution of sorghum phenolic compounds to its antioxidant capacity [68,69]. The results indicate that the effects of unmalted cereals depend on the interaction between cereal type and inclusion level. Sorghum showed the greatest potential to enhance the antioxidant properties of craft Ale beer, 40% rice produced the greatest reduction in ethanol and antioxidant capacity, and the KI and amino acid profile were modified by the three cereals in a cereal-dependent manner.

### 2.7. Sensory Evaluation

The sensory evaluation (Table 7) revealed that the partial substitution of barley malt with unmalted rice, wheat, and sorghum significantly (*p* < 0.05) affected overall acceptability, sweetness, aroma, body, and carbonation, whereas sensory bitterness did not differ among formulations. These findings show that unmalted cereal adjuncts can influence consumer sensory perception, which is a key determinant of the quality and market acceptance of craft beers [70]. Overall acceptability differed significantly among the formulations: T5 (20% sorghum + 80% barley malt) received the lowest score (4.95), significantly lower than all the other formulations, which did not differ from each other, including T6 (40% sorghum + 60% barley malt; 6.32, the numerically highest) and the control (6.19). This finding is noteworthy because both T5 and T6 contained sorghum. The lower acceptability of T5 cannot be attributed to bitterness, since its sensory bitterness score (5.41) did not differ from those of the other formulations despite its markedly higher analytical bitterness (36.565 IBU). Instead, in T5 the lowest acceptability coincided with the lowest body score (4.93, significantly lower than the control), one of the lowest sweetness scores (4.14, shared with T2), and a low aroma score (5.12). No correlation analysis between the sensory attributes and acceptability was performed, so these coincidences should be considered only as possible associations. In contrast, the high acceptability of T6 indicates that a 40% sorghum substitution remained sensorially acceptable under the conditions studied. According to Bamforth [35], consumer preference for beer depends on the balance among sweetness, bitterness, and aroma rather than on the absolute intensity of any individual sensory attribute.

Sweetness differed significantly among treatments: T6 had the highest score (5.14), significantly higher than T2 (4.18) and T5 (4.14), which received the lowest ratings, whereas the control and the other formulations did not differ significantly from either group. The higher sweetness score of T6 could be related to the low diastatic power of sorghum, which may limit starch hydrolysis and result in a greater retention of non-fermentable carbohydrates [34]; however, residual extract and dextrins were not measured, and the data do not support this explanation consistently: T2, which had the lowest ethanol concentration (and would therefore be expected to retain more residual sugars if attenuation were the main driver), received the lowest sweetness score, and T6 had an ethanol concentration similar to that of T1 (Table 2). Likewise, the lower sweetness of T2 cannot be generalized to rice, since T1 did not differ from the control. Sweetness perception may depend on factors other than residual carbohydrates, which were not evaluated in this study.

Although considerable differences in instrumental bitterness (IBU) were observed among the formulations, sensory bitterness did not differ significantly among treatments. This apparent discrepancy is consistent with the fact that bitterness perception depends not only on iso-α-acid concentration but also on interactions involving ethanol, proteins, dextrins, and polyphenols [71], and that other beer components, such as carbonation and viscosity, can modify trigeminal stimulation [72]. Consequently, despite T5 exhibiting the highest analytical IBU value, its perceived bitterness was not significantly different from that of the control beer. These findings highlight the importance of distinguishing between analytical bitterness and sensory bitterness. Significant differences were also observed for aroma: the control beer and T2 received the highest aroma scores (5.90 and 5.93), whereas T4 and T6 obtained the lowest ratings (5.02 and 5.03). The high aroma score of T2 cannot be explained by the higher alcohols measured, since T2 had the lowest isoamyl alcohol concentration (0.014%) and an isobutanol concentration similar to that of the other beers (Table 2). Aroma-active compounds other than these alcohols, such as esters, were not determined, so the aroma differences remain unexplained by the present chemical data.

Body was highest in the control beer (5.71) and lowest in T5 (4.93), which was the only formulation significantly lower than the control; the remaining adjunct beers did not differ significantly from the control. Beer body is associated with the concentration of residual dextrins, soluble proteins, β-glucans, and polysaccharides that contribute to viscosity and mouthfeel [35]. Sorghum contained relatively high dietary fiber levels (Table 1), and part of this fiber may consist of insoluble fractions that contribute little to sensory viscosity [38]; however, the low body score was observed in T5 but not in T6, and these compounds were not measured in the beers. Carbonation also differed significantly among treatments, with T1 receiving the highest score (6.42) and T3 the lowest (5.38), whereas T2, T4, T5, and T6 did not differ from the control.

Carbonation perception depends on carbon dioxide production and release as well as on beer viscosity and foam stability [73]. In this study, carbonation was obtained by bottle conditioning with 5 g/L of brown sugar, and carbon dioxide content, viscosity, and foam were not measured; therefore, the differences in perceived carbonation (higher in T1 and lower in T3) cannot be attributed to a specific mechanism, and they were not consistent within each cereal (T2 and T4 did not differ from the control). Overall, the sensory results show that unmalted cereal adjuncts modified some attributes of Ale beer in a formulation-dependent manner: 40% rice (T2) received one of the highest aroma scores together with a lower sweetness score; 20% rice (T1) received the highest carbonation score; the wheat formulations did not differ from the control in overall acceptability, sweetness, or body, although T3 had lower carbonation and T4 lower aroma scores; and sorghum produced contrasting responses, with T5 showing the lowest acceptability and body and T6 an acceptability comparable to the control. When the antioxidant and sensory results are considered together, 40% sorghum (T6) combined the highest antioxidant capacity with an overall acceptability comparable to that of the control under the conditions studied. These findings agree with those reported by Šimečková et al. [74], who concluded that the successful application of alternative cereals in brewing depends on achieving an appropriate balance among chemical composition, fermentation performance, and consumer sensory acceptance.

Regarding the potential influence of consumers on the sensory evaluation, the ANOVA performed under the randomized complete block design (RCBD) showed no significant effect of consumer on any of the sensory attributes evaluated (*p* > 0.05; Table S1).

### 2.8. Principal Component Analysis

**Figure 1:**
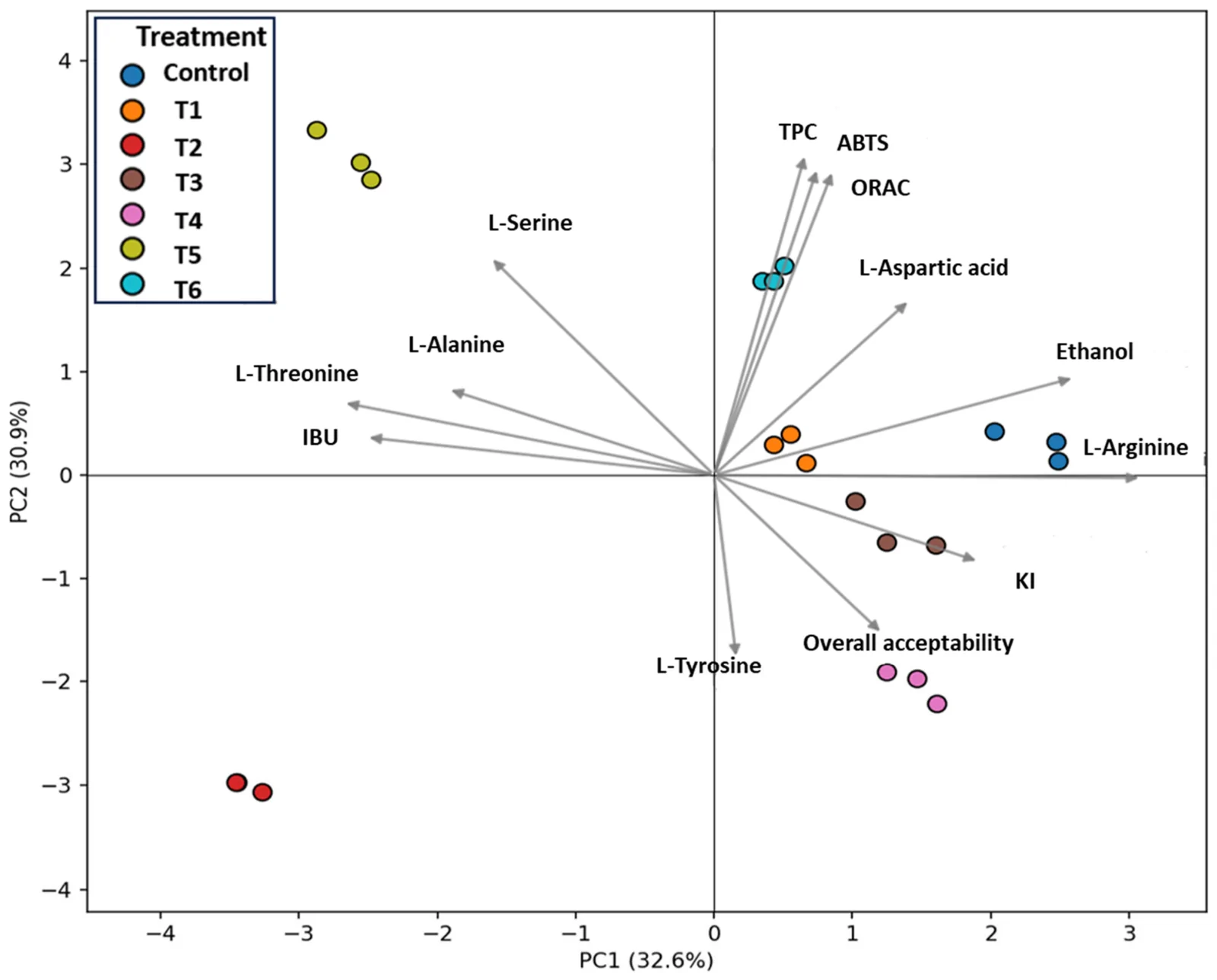
Principal component analysis (PCA) biplot of craft Ale beers formulated with different unmalted cereal adjuncts.

PCA was performed to explore the relationships among ethanol, nitrogen-related parameters, amino acid composition, antioxidant properties, bitterness, and overall sensory acceptability of Ale beers produced with different proportions of unmalted cereal adjuncts (Figure 1); isobutanol and isoamyl alcohol were not included in the PCA. The first two principal components explained 63.5% of the total variance, with PC1 and PC2 accounting for 32.6% and 30.9%, respectively. The PCA showed a clear separation among the formulations, in which the three batches of each formulation grouped together, indicating that both the type and the proportion of the unmalted cereal influenced the chemical and sensory profile of the beers. This finding is consistent with previous studies showing that the incorporation of unmalted cereal adjuncts can modify wort composition, fermentation performance, and the analytical and sensory characteristics of beer [13,17,23].

The most pronounced separation was observed for the sorghum-containing beers. T6, formulated with 40% unmalted sorghum, was positioned in the same direction as TPC, ORAC, and ABTS, indicating a strong association with the antioxidant-related variables. This pattern was supported by the univariate results, as T6 exhibited the highest TPC (316.66 µg GAE 100 mL^−1^) and ORAC value (11.594 mmol TE L^−1^), together with a high ABTS value (7.302 mmol TE L^−1^, not different from that of T5). T5, containing 20% sorghum, was located in a different region of the multivariate space (negative PC1 and positive PC2), closer to the direction of L-serine and, to a lesser extent, L-threonine, L-alanine, and IBU, although it also showed elevated TPC and ABTS. These results suggest that, in the multivariate space, increasing the proportion of unmalted sorghum from 20% to 40% shifted the beer profile toward the antioxidant variables. Sorghum is recognized as a cereal with a distinctive phytochemical composition and considerable potential as an alternative brewing material [14,24,44,47], and its phenolic compounds have been associated with substantial antioxidant activity [47,62].

The close orientation of TPC, ORAC, and ABTS further suggests a positive multivariate relationship between phenolic content and antioxidant capacity. Similar associations have been reported in different beer types, where phenolic compounds contribute substantially to antioxidant properties, although the antioxidant capacity of beer may also depend on other compounds and interactions within the beer matrix [18,55,71,75]. Therefore, the antioxidant behavior observed for the sorghum-containing formulations cannot be attributed exclusively to total phenolic content, but rather to the combined contribution of multiple antioxidant compounds present in the final beer.

The rice-containing treatments showed a clear concentration-dependent separation. T1, containing 20% unmalted rice, was located near the origin on the positive side of PC1, in the direction of the control, whereas T2, containing 40% rice, was clearly separated toward the negative side of both PC1 and PC2, opposite to the TPC, ORAC, ABTS, and ethanol vectors. This displacement was consistent with the analytical results, as T2 exhibited the lowest TPC, ORAC, ABTS, and ethanol values among all formulations. Previous research has shown that the incorporation of rice as an adjunct can modify extract composition, fermentation performance, alcohol production, and antioxidant characteristics depending on the type and proportion of rice used [13,21,23]. Thus, the separation between T1 and T2 indicates that increasing the proportion of unmalted rice from 20% to 40% substantially modified the chemical profile of the resulting beer.

The wheat-containing treatments (T3 and T4) occupied a distinct region of the multivariate space, on the positive side of PC1 and the negative side of PC2, in the direction of KI and, in the case of T4, of overall acceptability and L-tyrosine. The separation between T3 and T4 along PC2 suggests that the effect of unmalted wheat depended on the substitution level. Changes in protein composition and degradation during brewing can influence the release of nitrogenous compounds in wort and, consequently, yeast metabolism and the composition of the final beer [37,48], and the KI is associated with the degree of protein modification and nitrogen solubilization in brewing materials [76]; in this study, KI increased from T3 to T4 (Table 3).

The distribution of amino acids and ethanol in the PCA indicates that nitrogen-related variables contributed to the differentiation of the formulations. L-Serine, L-threonine, and L-alanine were oriented toward the negative side of PC1, together with IBU, in the region of T5, whereas L-arginine and ethanol were oriented toward the control. FAN and individual amino acids are important nutritional sources for yeast and can influence its metabolic activity [48]; the positioning of the treatments according to their amino acid profiles may therefore reflect differences in substrate composition resulting from the replacement of barley malt with different unmalted cereals, as well as differences in yeast utilization, which cannot be distinguished here. Because higher alcohols were not included in the PCA, this analysis cannot be used to evaluate the relationship between amino acid catabolism through the Ehrlich pathway and higher alcohol formation.

The control beer was positioned closer to ethanol and L-arginine, which was consistent with its significantly higher ethanol content (8.758%) compared with all the adjunct-containing formulations. Partial replacement of barley malt with unmalted cereals can modify the availability of fermentable carbohydrates, enzymatic activity, and nitrogen compounds, thereby influencing yeast metabolism and ethanol production [28,32,73]. The separation of the control from the adjunct-containing treatments therefore reflects the influence of cereal substitution on the final chemical composition of the beers.

The PCA also provides information regarding the relationship between antioxidant properties and sensory quality. Although T5 showed elevated TPC and ABTS, it exhibited the lowest overall acceptability among the formulations. In contrast, T6 combined the strongest antioxidant profile with an overall acceptability value (6.32) statistically comparable to that of the control and the other formulations, except T5. This finding indicates that the relationship between antioxidant enhancement and sensory acceptance was dependent on the specific formulation rather than following a simple inverse relationship. Previous studies have shown that cereal adjuncts can influence both the nutritional and sensory properties of beer, while phenolic compounds may also contribute to sensory attributes such as bitterness, astringency, and other flavor characteristics [13,19,70].

The PCA indicated that both the type and the proportion of unmalted cereal adjuncts influenced the global characteristics of the Ale beers. Rice, wheat, and sorghum generated distinct multivariate profiles associated with differences in antioxidant capacity, amino acid composition, ethanol, bitterness, and sensory acceptability. Among the evaluated formulations, the incorporation of 40% unmalted sorghum (T6) stood out because it produced the strongest antioxidant profile while maintaining overall acceptability comparable to that of the control, although with a lower ethanol content. These results indicate the potential of unmalted sorghum as an adjunct for the development of craft beers with enhanced antioxidant capacity without compromising overall acceptability under the conditions studied.

Several limitations should be considered when interpreting these findings. The study included a limited number of independent beer formulations, which restricted the statistical power for more complex multivariate analyses and supported the exploratory use of PCA. In addition, the total phenolic content provides an overall estimate of phenolic compounds and does not allow the individual contribution of specific phenolic sources to be quantitatively distinguished. Matrix-dependent effects during brewing may also have contributed to the observed differences. Future studies should therefore include larger experimental designs and targeted chromatographic characterization of individual phenolic compounds, together with supervised multivariate approaches when a sufficient number of independent observations is available.

## 3. Materials and Methods

### 3.1. Raw Materials

Pale Ale barley malt (*Hordeum vulgare*; Bestmalz, Heidelberg, Germany) was used as the base malt in all brewing trials. The brewing yeast was *S. cerevisiae* SafAle US-05 (Fermentis, Marcq-en-Barœul, France), while Cascade hops (*Humulus lupulus*; YHK, Ontario, CA, USA) were employed as the bittering hop. Unmalted rice (*Oryza sativa*), wheat (*Triticum aestivum*), and sorghum (*Sorghum bicolor*) were obtained from local producers in La Perla Market (Nuevo Chimbote, Ancash, Peru) and used as adjunct grains to partially replace barley malt. Food-grade gelatin (Universal, Lima, Peru) was added as a fining agent during wort boiling, whereas brown sugar (San Jacinto, Peru) was used for bottle conditioning and natural carbonation.

### 3.2. Physicochemical Characterization

The physicochemical composition of barley malt and adjunct grains (rice, wheat, and sorghum) was determined prior to brewing. Moisture, crude protein, crude fat, crude fiber, and ash contents were analyzed according to AOAC Official Methods 950.46, 987.13, 2003.05, 962.09, and 942.05, respectively [77]. The TDF was determined using an enzymatic-gravimetric method according to AOAC Official Method 985.29 [78].

Total carbohydrate content was calculated by difference [79], using the following Equation (1):

Carbohydrates (%) = 100 − [Moisture (%) + Protein (%) + Fat (%) + Ash (%)](1)

### 3.3. Brewing Process

Ale beers were produced using Pale Ale malt to obtain an original wort of 19°Bx and a target bitterness of 20 IBU using Cascade hops. Fermentation was carried out with SafAle US-05 yeast, and each brewing batch consisted of 50 L of wort. Rice, wheat, and sorghum were incorporated as unmalted adjunct grains at substitution levels of 20% and 40% of the malt grist (Table 8). These substitution levels were selected to represent moderate and higher degrees of unmalted cereal incorporation, respectively, within the same barley malt-based brewing framework. Previous brewing studies have evaluated adjunct incorporation at moderate levels, including approximately 20%, to assess their effects on wort and beer characteristics, while the use of up to 40% unmalted cereals has also been reported as technically feasible in brewing [15,24,25]. Therefore, the selected levels allowed the effects associated with increasing adjunct incorporation to be evaluated across rice, wheat, and sorghum under comparable brewing conditions.

For each formulation, three independent brewing batches (*n* = 3) were produced, each with a 50 L wort volume. The replicates corresponded to independent production batches, processed separately from milling through bottling, and the samples obtained were used for physicochemical, compositional, and instrumental analyses.

Barley malt was milled using a two-roller mill adjusted to a 0.5 mm roller gap, whereas rice, wheat, and sorghum were milled separately using a manual grain mill with an average particle size of approximately 2.0 mm. The grist was mashed in stainless steel vessels using brewing water preheated to 70 °C. Mashing was conducted for 60 min. Wort recirculation was subsequently performed using a stainless-steel magnetic pump equipped with a sparging arm until a clear wort was obtained. Batch sparging was carried out with water maintained at 70 °C to recover the remaining extract from the spent grains. The collected wort was boiled for 60 min at 100 °C. Cascade hops were added at the beginning of the boiling stage to provide bitterness, while food-grade gelatin was incorporated after 45 min as a fining agent. At the end of boiling, whirlpooling was performed for 10 min to facilitate trub separation. The clarified wort was cooled to approximately 25 °C using a stainless-steel plate heat exchanger before being transferred into a temperature-controlled stainless-steel conical fermenter. Primary fermentation was conducted at 20 ± 0.5 °C for seven days under automatic temperature control using a glycol chiller system. Subsequently, beers were cold conditioned at 7 °C for an additional seven days to promote yeast sedimentation and improve clarity. Following cold conditioning, sedimented yeast was removed through the bottom valve of the fermenter. The beer was transferred into a sanitized stainless-steel vessel and primed with 5 g/L of brown sugar for natural carbonation. Finally, beers were bottled in 330 mL amber glass bottles, crown-capped, and conditioned at room temperature in darkness for seven days before physicochemical and sensory analyses.

### 3.4. Determination of Volatile Alcohols by Gas Chromatography

Volatile alcohols, including ethanol, isobutanol, and isoamyl alcohol, were determined using a Shimadzu GC-2010 gas chromatograph (Shimadzu Corporation, Kyoto, Japan) equipped with a flame ionization detector (FID). Chromatographic separation was performed using a Stabilwax-DA fused-silica capillary column (Restek, Bellefonte, PA, USA; 30 m × 0.25 mm i.d., 0.50 μm film thickness; Cat. No. 11038).

Before chromatographic analysis, beer samples were degassed in an ultrasonic cleaner (Cole-Parmer, Model 8892-26, Series QPC010698219F, Vernon Hills, IL, USA) for 5 min at 20–25 °C to remove dissolved carbon dioxide. The bath temperature was monitored to minimize sample heating and potential losses of volatile compounds. The degassed samples were subsequently centrifuged using a refrigerated centrifuge (Sigma, 4-16K, Rödermark, Germany) at 4 °C for 10 min to remove suspended particles and residual yeast cells. The resulting supernatants were collected for chromatographic analysis. Samples containing analyte concentrations above the applicable calibration range were diluted with ultrapure water, and the corresponding dilution factors were considered in the final concentration calculations.

A 1 μL aliquot of each prepared sample was injected in split mode at a split ratio of 300:1, with the injector maintained at 200 °C. The oven temperature was initially set at 55 °C and held for 3 min, followed by a temperature ramp of 10 °C min^−1^ to 120 °C, which was maintained for an additional 2 min. Helium was used as the carrier gas at a constant flow rate of 1.41 mL min^−1^, corresponding to a linear velocity of 32.9 cm s^−1^. The purge flow was set at 3.0 mL min^−1^. The FID temperature was maintained at 200 °C, with helium, hydrogen, and air flow rates of 30, 40, and 400 mL min^−1^, respectively.

Quantification was performed using the external standard method. Individual calibration series were prepared for ethanol, isobutanol, and isoamyl alcohol using the corresponding analytical standards. The external calibration standards were prepared by diluting the analytical standards in ultrapure water. The calibration levels for ethanol were 0.020, 2, 4, 6, 8, and 10% (*v v*^−1^), whereas those for isobutanol and isoamyl alcohol were 0.020, 0.5, 2, 4, 6, and 10% (*v v*^−1^). A separate low-concentration calibration series comprising 0.0005, 0.001, 0.002, 0.005, 0.010, 0.015, and 0.020% (*v v*^−1^) was used to estimate the limits of detection (LOD) and quantification (LOQ). Calibration curves were constructed by plotting chromatographic peak area against analyte concentration, and the corresponding regression equations were used for concentration calculations. Compound identification was based on comparing the retention times of sample peaks with those of the corresponding analytical standards analyzed under identical chromatographic conditions. Calibration performance was characterized by linear regression according to Equation (2):

(2) y = mx + b

where *y* represents the chromatographic peak area, *x* is the analyte concentration, *m* is the slope, and *b* is the intercept. The coefficient of determination (R^2^) and the residual standard deviation of the regression (S_γ_/_x_) were recorded. The LOD and LOQ were estimated from the low-concentration calibration data using Equations (3) and (4), respectively:

(3) $$\mathrm{LOD}\;=\;\frac{\left(3.3\;\times \;S_{\gamma /x}\right)}{m}$$

(4) $$\mathrm{LOQ}=\frac{\left(10\times S_{\gamma /x}\right)}{m}$$

The potential matrix effect (ME) was evaluated by comparing the slopes of matrix-matched and solvent-based calibration curves and was expressed according to Equation (5). The solvent-based calibration standards were prepared in ultrapure water.

For matrix-matched calibration, the control beer was used as the initial matrix, and known amounts of the corresponding alcohol standards were added to prepare new calibration curves at six concentration levels for each analyte. For ethanol, the nominal concentration levels added were 0.02, 2, 4, 6, 8, and 10% (*v v*^−1^), whereas for isobutanol and isoamyl alcohol, the corresponding levels were 0.02, 0.5, 2, 4, 6, and 10% (*v v*^−1^). Before fortification, the control beer was diluted with ultrapure water to obtain a matrix containing 0.8% (*v v*^−1^) ethanol. The initial concentrations of isobutanol and isoamyl alcohol in the undiluted control beer were 0.187% (*v v*^−1^) and 0.022% (*v v*^−1^), respectively. These background concentrations were therefore present in the beer matrix before the corresponding standards were added. The analytical responses obtained from the fortified beer matrices were used to construct the matrix-matched calibration curves and were compared with those obtained from the calibration curves prepared in ultrapure water.

(5) $$ME\;(\%)=\frac{m_{matrix}}{m_{solvent}}\times 100$$

where $m_{matrix}$ and $m_{solvent}$ represent the slopes of the matrix-matched and solvent-based calibration curves, respectively.

Method recovery was assessed by fortifying a control beer sample with a known amount of each alcohol, followed by a new chromatographic analysis. The control beer sample was fortified by adding a known amount of the target alcohol, and the fortified sample was analyzed under the same chromatographic conditions as the unfortified control. Recovery was calculated according to Equation (6):

(6) $$\mathrm{Recovery}\;(\%)\;=\;\frac{C_{fortified}-C_{control}}{C_{added}}\times 100$$

where $C_{fortified}$ is the measured concentration in the fortified sample, $C_{control}$ is the concentration measured in the unfortified control, and $C_{added}$ is the concentration added during fortification. The nominal fortification level was 0.5% (*v v*^−1^). The calibration parameters and analytical validation parameters are reported in Table S2.

### 3.5. International Bitterness Units

IBU were determined according to the ASBC Beer-23A method [80,81]. Beer samples (5 mL) were acidified with 0.5 mL of 3 N HCl and extracted with 10 mL of isooctane. After mixing and centrifugation (3000 rpm, 5 min), the organic phase was recovered, and its absorbance was measured at 275 nm using a UV–Vis spectrophotometer (Jasco, V-670, Tokyo, Japan). The spectrophotometrically determined bitterness was expressed as IBU, calculated by multiplying the absorbance value at 275 nm by a factor of 50.

### 3.6. Kolbach Index

The KI was calculated as the ratio of total soluble nitrogen (TSN) in the wort to total nitrogen (TN) in the malt according to the European Brewery Convention (EBC) Method 4.9.1. TN was determined by the Dumas combustion method (EBC Method 4.3.2), whereas soluble nitrogen was measured in the congress wort following the corresponding EBC analytical procedure [82,83]. The KI was calculated as Equation (7):

(7) $$KI\left(\%\right)=\frac{TSN}{TN}\times \;100$$

### 3.7. Total Phenolic Content

The TPC of the beer samples was determined using the Folin–Ciocalteu assay, adapted from Saura-Calixto et al. [84]. The assay provides an estimate of the total phenolic content expressed as gallic acid equivalents (GAE), rather than a direct measurement of individual phenolic compounds. A gallic acid stock solution (450 μg mL^−1^) was prepared by dissolving 0.0225 g of gallic acid (C_7_H_6_O_5_) in 50 mL of distilled water. A 20% sodium carbonate (Na_2_CO_3_) solution was prepared by dissolving 2 g of Na_2_CO_3_ in 10 mL of distilled water, followed by sonication for 5 min. A 2 N Folin–Ciocalteu reagent was used.

Gallic acid standard solutions at concentrations of 7.2, 14.4, 21.6, 28.8, and 36.0 μg mL^−1^ were prepared from the stock solution to construct the calibration curve. Appropriate aliquots of each standard solution were mixed with 100 μL of 2 N Folin–Ciocalteu reagent and allowed to react for 5 min. Subsequently, 50 μL of 20% Na_2_CO_3_ solution was added, and the mixtures were brought to the final reaction volume with distilled water. After incubation for 1 h, 200 μL of each reaction mixture was transferred to a microplate well, and the absorbance was measured at 730 nm using a Synergy^TM^ H1 multimode microplate reader (BioTek Instruments, Inc., Cheadle, UK).

The calibration curve was described by the equation Y = 0.0567X − 0.0664 (R^2^ = 0.994), where X represents the gallic acid concentration (μg mL^−1^) and Y represents the absorbance.

Before analysis, the beer samples were degassed to remove dissolved carbon dioxide. Subsequently, 800 μL of degassed beer was transferred to an Eppendorf tube and mixed with 100 μL of 2 N Folin–Ciocalteu reagent. After standing for 5 min, 50 μL of 20% Na_2_CO_3_ solution and 300 μL of distilled water were added. The reaction mixture was then incubated in the dark for 1 h at room temperature. Subsequently, 200 μL of the reaction mixture was transferred to a microplate well, and the absorbance was measured at 730 nm using a Synergy^TM^ H1 multimode microplate reader (BioTek Instruments, Inc., Cheadle, UK).

The TPC was calculated from the gallic acid calibration curve. When the absorbance of a beer sample fell outside the linear range of the calibration curve, the degassed beer sample was diluted 1:2 with distilled water before the addition of the Folin–Ciocalteu reagent. The diluted sample was then reanalyzed following the same procedure described above, and a dilution factor of 2 was applied to the calculated TPC value to account for the dilution and express the result relative to the original beer sample. The results were expressed as μg of gallic acid equivalents per 100 mL of beer (μg GAE 100 mL^−1^). All measurements were performed in triplicate, and the results were expressed as the mean ± standard deviation.

### 3.8. Antioxidant Capacity

#### 3.8.1. Oxygen Radical Absorbance Capacity Assay

The ORAC assay was performed according to AOAC Official Method 2012.23 [85], with minor modifications. The analysis was carried out using a Synergy^TM^ H1 multimode microplate reader (BioTek Instruments, Inc., Cheadle, UK) equipped with excitation and emission wavelengths of 485 nm and 530 nm, respectively, and with temperature control maintained at 37 °C. Beer samples were centrifuged at 14,000× *g* for 15 min, and the supernatants were appropriately diluted with 75 mM potassium phosphate buffer (pH = 7.4) prior to analysis. Trolox (6-hydroxy-2,5,7,8-tetramethylchroman-2-carboxylic acid) was used as the antioxidant standard, and calibration solutions ranging from 0 to 160 μM were prepared in phosphate buffer. The reaction mixture consisted of 150 μL of fluorescein working solution (0.1112 μM), 25 μL of the diluted beer sample or Trolox standard, and 25 μL of freshly prepared AAPH (2,2′-azobis(2-amidinopropane) dihydrochloride), resulting in a final reaction volume of 200 μL per well. After the addition of fluorescein and samples, the microplate was incubated at 37 °C for 30 min. Subsequently, the AAPH solution was rapidly added to initiate peroxyl radical generation, and fluorescence decay was automatically monitored every 1 min for 35 min. Antioxidant activity was determined from the net area under the fluorescence decay curve (Y = net Area Under the Curve) after subtracting the blank response. A calibration curve was constructed using Trolox concentrations (X), which exhibited excellent linearity (R^2^ = 0.999). Finally, ORAC values were calculated using the regression equation of the calibration curve (Y = 0.135X + 0.501) and expressed as millimoles of Trolox equivalents per liter of beer (mmol TE L^−1^).

#### 3.8.2. 2,2′-Azino-bis (3-Ethylbenzothiazoline-6-sulfonic Acid) Assay

The antioxidant capacity of beer samples was determined using the 2,2′-azino-bis (3-ethylbenzothiazoline-6-sulfonic acid) radical cation (ABTS^•+^) decolorization assay according to the method of Re et al. [61], with minor modifications for microplate analysis. The ABTS radical cation was generated by reacting 7.0 mM ABTS (Sigma-Aldrich, St. Louis, MO, USA) with 2.45 mM potassium persulfate (K_2_S_2_O_8_) in a 1:1 (*v v*^−1^) ratio. The reaction mixture was incubated in the dark at room temperature (22–25 °C) for 16 h to allow complete radical formation. Prior to analysis, the ABTS^•+^ solution was diluted with phosphate-buffered saline (PBS, pH = 7.4) until an absorbance of 0.70 ± 0.02 at 734 nm was obtained. Beer samples were previously degassed by sonication and appropriately diluted with distilled water when necessary. For the assay, 10 µL of sample or Trolox standard solution was mixed with 190 µL of the diluted ABTS^•+^ working solution in 96-well microplates. The reaction mixture was incubated for 6 min at room temperature in the dark, and the decrease in absorbance was measured at 734 nm using a Synergy^TM^ H1 multimode microplate reader (BioTek Instruments, Inc., Cheadle, UK).

Trolox was used as the reference antioxidant at concentrations ranging from 20 to 800 µmol L^−1^. The calibration curve was constructed from the percentage inhibition values according to the linear regression equation: Y = 6.2284X + 0.1084 (R^2^ = 0.994), where Y represents the percentage inhibition of the ABTS radical and X corresponds to the antioxidant capacity expressed as millimoles of Trolox equivalents per liter of beer (mmol TE L^−1^). The percentage inhibition of the ABTS radical was calculated using the following Equation (8):

(8) $$ABTS\;inhibition\;\left(\%\right)=\;\frac{\left(A_{0}-\;A_{s}\right)}{A_{0}}\;\times 100$$

where A_0_ is the absorbance of the ABTS radical solution (blank) and A_s_ is the absorbance of the sample after the reaction.

### 3.9. Determination of Free Amino Acid Content by Liquid Chromatography

Free amino acids were quantified by liquid chromatography–electrospray ionization–mass spectrometry (LC–ESI–MS) using an Agilent 1290 Infinity III system (Agilent Technologies, Santa Clara, CA, USA) equipped with an InfinityLab Poroshell 120 EC-C18 column (3.0 × 150 mm, 1.9 μm). Chromatographic separation was performed using gradient elution with mobile phase A (acetonitrile/water, 95:5, *v*/*v*, containing 5 mM ammonium formate, pH 3.0) and mobile phase B (water/acetonitrile, 70:30, *v v*^−1^, containing 5 mM ammonium formate, pH 3.0). The gradient started at 80% A and 20% B, reached 50% A and 50% B at 10 min, and subsequently returned to the initial conditions. The total analysis time was 18 min. The flow rate was 0.4 mL min^−1^, the column temperature was maintained at 40 °C, and the injection volume was 1 μL. Mass spectrometric detection was performed using an electrospray ionization (ESI) source in positive ion mode, with a gas temperature of 325 °C, a capillary voltage of 3500 V, and a scan range of *m*/*z* 50–300. Amino acids were quantified in selected ion recording (SIR) mode by monitoring their characteristic ions.

Calibration standards were prepared at five concentration levels (10, 25, 100, 250, and 1000 μg mL^−1^) and analyzed to establish individual calibration curves for each amino acid. Calibration linearity was assessed using the coefficient of determination (R^2^) and the residual standard deviation of the regression (S_γ_/_x_). The limits of detection (LOD) and quantification (LOQ) were estimated from the low-concentration calibration data using the same regression-based approach described for the gas chromatography method, as defined by Equations (3) and (4), respectively. The corresponding precursor ions, optimized fragmentor voltages, calibration equations, R^2^ values, LODs, and LOQs are reported in Supplementary Table S3.

The potential matrix effect was evaluated by fortifying beer samples with the amino acid standards at 100 μg mL^−1^ and comparing the slopes of the matrix-matched and solvent-based calibration curves. The matrix effect was expressed as the ratio of the matrix-matched calibration slope to the corresponding solvent-based calibration slope, multiplied by 100, according to Equation (5). Values close to 100% indicate negligible differences between the two slopes, whereas values below or above 100% indicate potential signal suppression or enhancement, respectively. Method recovery was assessed by comparing the measured amino acid concentrations in fortified and unfortified beer samples, with recovery calculated according to Equation (6). The calibration and analytical validation parameters are summarized in Supplementary Table S3.

### 3.10. Sensory Evaluation

Sensory analysis was conducted with 100 untrained consumers who reported regularly consuming beer. The consumers were recruited in Nuevo Chimbote, Ancash Region, Peru, and included participants aged 18–50 years, with an equal distribution by sex (50% women and 50% men). The consumers were randomly divided into three groups to evaluate samples from the three independent brewing batches. The first group comprised 33 consumers who evaluated all seven beer formulations (Control, T1, T2, T3, T4, T5, and T6) prepared in Brewing Batch 1; the second group comprised 33 consumers who evaluated the same seven formulations prepared in Brewing Batch 2; and the third group comprised 34 consumers who evaluated the seven formulations prepared in Brewing Batch 3. Each consumer evaluated the seven formulations corresponding to only one assigned brewing batch and did not evaluate samples from the other two batches.

Beer samples were refrigerated (6.0 ± 0.5 °C) and served in 10 mL portions in transparent cups identified with randomly assigned three-digit codes. The presentation order of the samples was randomized to minimize potential order effects. Carbonated water was provided between samples to minimize carryover effects.

The sensory attributes evaluated included sweetness, bitterness, aroma, body, carbonation, and overall acceptability. Each attribute was evaluated using a 7-point continuous hedonic scale, in which consumers indicated their level of acceptance by marking a position along a continuous line. The scale was numerically represented from 1 to 7, where 1 corresponded to “dislike extremely,” 4 to “neither like nor dislike,” and 7 to “like extremely.”

Prior to participation, all consumers provided informed consent and were informed about the purpose and procedures of the sensory evaluation, as well as the confidentiality of their data. The sensory study was conducted with the approval of the Ethics Committee of the Universidad Nacional del Santa (CEI-UNS-PI-02-2026).

The sensory experiment was analyzed using a randomized complete block design (RCBD), in which beer formulation was considered the treatment factor and each consumer was considered a block. Because each consumer evaluated all seven formulations, the complete-block structure allowed individual differences in consumer perception and scoring to be accounted for in the analysis. Differences among beer formulations were evaluated using analysis of variance (ANOVA), followed by Tukey’s honestly significant difference (HSD) test when significant effects were detected at a significance level of *p* < 0.05. Sensory results were expressed as mean ± standard deviation.

### 3.11. Statistical Analysis

For the physicochemical, antioxidant, and compositional analyses of the produced beers, including the KI, IBU, TPC, ABTS, ORAC, free amino acids, and higher alcohols, the effects of unmalted cereal adjuncts were evaluated using a two-factor factorial design (3 × 2). The first factor was cereal type, with three levels (rice, wheat, and sorghum), while the second factor was substitution level, with two levels (20% and 40%). Therefore, the factorial analysis included treatments T1–T6 and was performed using a two-way analysis of variance (ANOVA) to evaluate the main effects of cereal type and substitution level, as well as their interaction.

Additionally, to compare all beer formulations, including the control beer produced with 100% malted barley, a completely randomized design (CRD) with a single factor was applied, consisting of seven formulation levels (Control and T1–T6). Differences among treatment means were evaluated using a one-way ANOVA, followed by Tukey’s honestly significant difference (HSD) test when significant differences were detected at a significance level of *p* < 0.05. The results of the physicochemical, antioxidant, and compositional analyses were expressed as the mean ± standard deviation based on three independent brewing batches per formulation. All statistical analyses were performed using Minitab Statistical Software (version 22; Minitab LLC., State College, PA, USA).

A principal component analysis (PCA) was performed using Python version 3.12.3 (Python Software Foundation, DE, USA) in a Linux environment to evaluate the multivariate relationships among the beer treatments and determine the variables contributing to their differentiation. The PCA included 21 observations corresponding to seven formulations (Control and T1–T6), with three observations per formulation. Each observation represented one independent brewing batch. Thus, the analytical measurements (TPC, ORAC, ABTS, free amino acids, KI, IBU, and ethanol) were retained at the brewing-batch level rather than being averaged across the three batches.

Thirteen variables were included: TPC, ORAC, ABTS, L-serine, L-arginine, L-aspartic acid, L-threonine, L-alanine, L-tyrosine, Kolbach index (KI), IBU, ethanol, and overall acceptability. Variable selection was based on data completeness, analytical reliability, and the relevance of the information represented. For the free amino acid profile, only amino acids with quantifiable concentrations across all treatments were included. Overall acceptability was selected as the sensory variable because it provided an overall measure of consumer perception. Ethanol was retained as the representative alcohol-related variable because the concentrations of the other volatile compounds were very low.

Overall acceptability was also incorporated into the PCA at the brewing-batch level. For each formulation, a separate mean overall acceptability score was calculated for each of the three independent brewing batches using the evaluations provided by the consumers assigned to that batch. Specifically, the mean for Batch 1 was calculated from 33 consumers, the mean for Batch 2 from 33 consumers, and the mean for Batch 3 from 34 consumers. Each consumer evaluated all seven formulations from only one assigned brewing batch. The three batch-specific overall acceptability means for each formulation were then paired with the corresponding analytical measurements from the same brewing batches. Consequently, the PCA comprised 21 observations (seven formulations × three independent brewing batches), with one batch-specific overall acceptability value per observation. This procedure preserved the correspondence between the sensory and analytical datasets at the brewing-batch level.

The dataset contained no missing values; therefore, no imputation or deletion of observations due to missing data was required. Due to differences in measurement scales, all variables were standardized using Z-scores (mean = 0 and standard deviation = 1). Subsequently, PCA was performed using the Pearson correlation matrix. The principal components were interpreted based on eigenvalues, the percentage of explained variance, and variable loadings. During the exploratory evaluation of configurations containing more than 13 variables, the cumulative percentage of variance explained by PC1 and PC2 was lower. The 13-variable configuration was therefore retained based on the combined consideration of data completeness, analytical reliability, variable relevance, and the variance explained by the first two principal components.

## 4. Conclusions

Partial replacement of barley malt with unmalted rice, wheat, and sorghum significantly modified the antioxidant capacity, ethanol and higher alcohol concentrations, nitrogen-related parameters, residual free amino acid profile, and sensory characteristics of Ale beer, and the effects varied according to cereal type and substitution level with significant cereal type × substitution level interactions observed for several response variables. All adjunct formulations produced less ethanol than the control, with the rice formulations showing particularly pronounced reductions. Isoamyl alcohol was lower in all adjunct beers, whereas isobutanol was practically unchanged. Among the formulations evaluated, the formulation containing the higher proportion of unmalted sorghum exhibited the highest measured total phenolic content and antioxidant capacity, although its ABTS response was not significantly different from that of the lower-sorghum formulation, and an overall acceptability comparable to that of the control, although with a lower ethanol content than the control; the lower-sorghum formulation showed lower sensory acceptability.

In contrast, the formulation containing the higher proportion of rice exhibited lower total phenolic content and antioxidant capacity, whereas the wheat formulations showed TPC and antioxidant capacity values similar to or lower than those of the control and higher ethanol concentrations than the rice and sorghum formulations. The differences in KI and residual amino acids were formulation-dependent and, because the wort composition was not analyzed, should be interpreted as formulation-associated changes rather than as evidence of specific biochemical mechanisms or of greater nitrogen utilization efficiency. PCA provided an exploratory visualization of associations among the measured physicochemical, antioxidant, nitrogen-related, ethanol, and sensory variables and showed differentiation among the beer formulations; however, given the limited number of independent brewing batches, these multivariate patterns should be interpreted cautiously and should not be considered evidence of causal relationships. Future studies should include larger experimental designs with a greater number of independent brewing batches, wort analysis (FAN and amino acids), targeted characterization of individual phenolic compounds, and supervised multivariate approaches, such as partial least squares (PLS), when a sufficient number of independent observations are available.

## Figures and Tables

**Table 1:** Proximate composition of the raw materials used for beer production.

Sample	Moisture (%)	Ash (%)	Fat (%)	Protein (%)	Carbohydrates (%)	TDF (%)
Barley	4.611 ± 0.101 ^b^	2.002 ± 0.025 ^b^	0.212 ± 0.018 ^b^	9.955 ± 0.120 ^c^	83.220 ± 0.222 ^a^	12.845 ± 0.384 ^b^
Wheat	12.544 ± 0.365 ^a^	1.920 ± 0.026 ^b^	0.133 ± 0.012 ^c^	10.245 ± 0.083 ^b^	75.158 ± 0.306 ^c^	6.938 ± 0.673 ^c^
Rice	12.196 ± 0.080 ^a^	0.374 ± 0.064 ^c^	0.359 ± 0.033 ^a^	8.362 ± 0.030 ^d^	78.708 ± 0.074 ^b^	8.743 ± 0.385 ^c^
Sorghum	12.583 ± 0.045 ^a^	2.652 ± 0.078 ^a^	0.136 ± 0.013 ^c^	11.126 ± 0.097 ^a^	73.502 ± 0.076 ^d^	30.431 ± 0.464 ^a^

Values are expressed as mean ± standard deviation (*n* = 3). Different lowercase superscript letters within the same column indicate significant differences among raw materials (Tukey’s honestly significant difference (HSD) test, *p* < 0.05). TDF: total dietary fiber.

**Table 2:** Alcohol composition of Ale beer formulations produced with different unmalted cereal adjuncts.

Formulation	Ethanol (%)	Isobutanol (%)	Isoamyl Alcohol (%)
Control	8.758 ± 0.199 ^a^	0.187 ± 0.005 ^a^	0.022 ± 0.005 ^a^
T1	6.473 ± 0.040 ^e^	0.183 ± 0.001 ^a^	0.016 ± 0.001 ^b^
T2	4.561 ± 0.020 ^f^	0.183 ± 0.001 ^a^	0.014 ± 0.000 ^b^
T3	8.112 ± 0.012 ^b^	0.184 ± 0.000 ^a^	0.015 ± 0.001 ^b^
T4	7.230 ± 0.049 ^c^	0.185 ± 0.001 ^a^	0.015 ± 0.001 ^b^
T5	6.805 ± 0.080 ^d^	0.182 ± 0.000 ^a^	0.015 ± 0.001 ^b^
T6	6.418 ± 0.067 ^e^	0.182 ± 0.000 ^a^	0.015 ± 0.001 ^b^

Different lowercase superscript letters within the same column indicate significant differences among beer formulations (Tukey’s honestly significant difference (HSD) test, *p* < 0.05). Values are expressed as mean ± standard deviation (*n* = 3), based on three independent brewing batches.

**Table 3:** Kolbach index (KI) and international bitterness units (IBU) of Ale beers produced with different unmalted cereal adjuncts.

Formulation	KI	IBU
Control	19.687 ± 1.992 ^a^	14.193 ± 0.081 ^e^
T1	18.090 ± 0.750 ^ab^	18.000 ± 0.014 ^d^
T2	14.716 ± 0.673 ^cd^	26.712 ± 0.378 ^b^
T3	16.514 ± 1.639 ^bc^	12.610 ± 0.014 ^g^
T4	19.827 ± 0.564 ^a^	23.425 ± 0.021 ^c^
T5	15.801 ± 0.453 ^bcd^	36.565 ± 0.085 ^a^
T6	13.341 ± 0.516 ^d^	13.313 ± 0.216 ^f^

Different lowercase superscript letters within the same column indicate significant differences among beer formulations (Tukey’s honestly significant difference (HSD) test, *p* < 0.05). KI: Kolbach index; IBU: international bitterness units. Values are expressed as mean ± standard deviation (*n* = 3), based on three independent brewing batches.

**Table 4:** Amino acid content in beer samples.

Amino Acid (µg mL^−1^)	Control	T1	T2	T3	T4	T5	T6
L-Serine	323.73 ± 0.82 ^d^	342.80 ± 0.61 ^b^	332.84 ± 0.13 ^c^	320.53 ± 0.48 ^e^	312.86 ± 0.52 ^f^	344.00 ± 0.85 ^b^	346.04 ± 0.71 ^a^
L-Arginine	169.04 ± 0.27 ^ab^	169.26 ± 0.13 ^a^	n.d.	168.86 ± 0.12 ^bc^	168.61 ± 0.03 ^c^	n.d.	169.32 ± 0.04 ^a^
L-Cysteine	244.86 ± 0.70 ^b^	247.97 ± 0.18 ^a^	n.d.	n.d.	n.d.	n.d.	n.d.
L-Aspartic acid	69.69 ± 0.22 ^b^	104.36 ± 0.55 ^ab^	n.d.	114.19 ± 0.07 ^a^	111.97 ± 0.23 ^a^	108.78 ± 0.48 ^a^	106.43 ± 0.02 ^a^
L-Threonine	243.57 ± 0.59 ^f^	250.05 ± 0.59 ^e^	260.21 ± 0.64 ^b^	252.78 ± 0.29 ^d^	254.01 ± 0.22 ^d^	264.45 ± 0.50 ^a^	258.70 ± 0.55 ^c^
L-Alanine	234.58 ± 0.22 ^ab^	236.69 ± 0.16 ^ab^	236.79 ± 0.12 ^a^	235.36 ± 0.23 ^ab^	230.27 ± 1.11 ^b^	237.27 ± 0.08 ^a^	233.26 ± 0.08 ^b^
L-Valine	34.31 ± 0.00	n.d.	n.d.	n.d.	n.d.	n.d.	n.d.
L-Isoleucine	80.86 ± 0.00	n.d.	n.d.	n.d.	n.d.	n.d.	n.d.
L-Leucine	40.61 ± 0.00 ^a^	n.d.	n.d.	n.d.	40.44 ± 0.22 ^a^	n.d.	n.d.
L-Tyrosine	n.d.	26.61 ± 0.01 ^a^	26.68 ± 0.01 ^a^	26.68 ± 0.02 ^a^	26.76 ± 0.08 ^a^	n.d.	26.68 ± 0.01 ^a^
L-Glycine	n.d.	n.d.	n.d.	74.21 ± 0.33	n.d.	n.d.	n.d.
L-Proline	n.d.	n.d.	n.d.	97.23 ± 1.72	n.d.	n.d.	n.d.

Values followed by the same lowercase letter within the same row are not significantly different (Tukey’s honestly significant difference (HSD) test, *p* > 0.05). n.d.: not detected < LOD (limit of detection) < LOQ (limit of quantification). Values are expressed as mean ± standard deviation (*n* = 3), based on three independent brewing batches.

**Table 5:** Total phenolic content and antioxidant capacity of Ale beers produced with different unmalted cereal adjuncts.

Formulation	TPC (µg GAE 100 mL^−1^)	ORAC (mmol TE L^−1^)	ABTS (mmol TE L^−1^)
Control	230.52 ± 6.23 ^d^	9.867 ± 0.316 ^b^	6.806 ± 0.016 ^b^
T1	260.18 ± 4.19 ^c^	6.903 ± 0.073 ^c^	6.647 ± 0.028 ^b^
T2	135.98 ± 1.77 ^f^	3.383 ± 0.081 ^e^	3.635 ± 0.085 ^d^
T3	239.43 ± 3.64 ^d^	6.543 ± 0.248 ^c^	5.235 ± 0.225 ^c^
T4	189.83 ± 5.74 ^e^	5.056 ± 0.079 ^d^	4.842 ± 0.154 ^c^
T5	277.89 ± 6.13 ^b^	10.216 ± 0.358 ^b^	7.638 ± 0.107 ^a^
T6	316.66 ± 4.59 ^a^	11.594 ± 0.322 ^a^	7.302 ± 0.267 ^a^

Different letters within the same column indicate significant differences (Tukey’s honestly significant difference (HSD) test, *p* < 0.05). TPC: total phenolic content; ORAC: oxygen radical absorbance capacity; ABTS: 2,2′-azinobis-(3-ethylbenzothiazoline-6-sulfonic acid); GAE: gallic acid equivalents; TE: trolox equivalents. Values are expressed as mean ± standard deviation (*n* = 3), based on three independent brewing batches.

**Table 6:** Factorial ANOVA and Tukey’s (HSD) comparison of beer characteristics according to cereal type and inclusion level.

Response Variable	Cereal (*p*)	Level (*p*)	Cereal × Level (*p*)	Rice 20%	Rice 40%	Wheat 20%	Wheat 40%	Sorghum 20%	Sorghum 40%
TPC (µg GAE 100 mL^−1^)	<0.001	<0.001	<0.001	260.179 ± 4.176 ^c^	135.984 ± 1.743 ^f^	239.433 ± 3.637 ^d^	189.827 ± 5.737 ^e^	277.886 ± 6.177 ^b^	316.665 ± 4.585 ^a^
ORAC (mmol TE L^−1^)	<0.001	<0.001	<0.001	6903.250 ± 72.966 ^a^	3382.617 ± 82.494 ^e^	6543.180 ± 245.736 ^a^	5055.667 ± 80.088 ^d^	10,215.500 ± 354.530 ^c^	11,593.967 ± 320.441 ^b^
ABTS (mmol TE L^−1^)	<0.001	<0.001	<0.001	6646.891 ± 28.058 ^c^	3635.294 ± 85.720 ^d^	5234.620 ± 226.793 ^b^	4841.803 ± 154.533 ^b^	7638.287 ± 106.228 ^a^	7301.586 ± 266.678 ^a^
L-Serine (mg mL^−1^)	<0.001	<0.001	<0.001	0.34283 ± 0.00060 ^a^	0.33287 ± 0.00015 ^c^	0.32057 ± 0.00050 ^d^	0.31287 ± 0.00055 ^e^	0.34400 ± 0.00085 ^a^	0.34607 ± 0.00072 ^b^
L-Arginine (mg mL^−1^)	<0.001	0.1089	<0.001	0.16927 ± 0.00012 ^a^	0.08430 ± 0.00000 ^b^	0.16887 ± 0.00015 ^c^	0.16860 ± 0.00000 ^d^	0.08430 ± 0.00000 ^b^	0.16933 ± 0.00006 ^a^
L-Aspartic acid (mg mL^−1^)	<0.001	<0.001	<0.001	0.10437 ± 0.00055 ^e^	0.03470 ± 0.00000 ^f^	0.11420 ± 0.00010 ^a^	0.11197 ± 0.00021 ^b^	0.10880 ± 0.00046 ^c^	0.10643 ± 0.00006 ^d^
L-Threonine (mg mL^−1^)	<0.001	<0.001	<0.001	0.25003 ± 0.00058 ^e^	0.26023 ± 0.00067 ^c^	0.25280 ± 0.00026 ^a^	0.25400 ± 0.00020 ^a^	0.26443 ± 0.00047 ^b^	0.25870 ± 0.00053 ^d^
L-Alanine (mg mL^−1^)	<0.001	<0.001	<0.001	0.23667 ± 0.00015 ^ab^	0.23680 ± 0.00010 ^a^	0.23537 ± 0.00023 ^b^	0.23030 ± 0.00113 ^d^	0.23730 ± 0.00010 ^a^	0.23327 ± 0.00012 ^c^
L-Tyrosine (mg mL^−1^)	<0.001	<0.001	<0.001	0.02660 ± 0.00000 ^c^	0.02670 ± 0.00000 ^a^	0.02670 ± 0.00000 ^a^	0.02677 ± 0.00006 ^b^	0.01330 ± 0.00000 ^d^	0.02670 ± 0.00000 ^a^
L-Cysteine (mg mL^−1^)	<0.001	<0.001	<0.001	0.24797 ± 0.00018 ^b^	0.00000 ± 0.00000 ^a^	0.00000 ± 0.00000 ^a^	0.00000 ± 0.00000 ^a^	0.00000 ± 0.00000 ^a^	0.00000 ± 0.00000 ^a^
L-Leucine (mg mL^−1^)	<0.001	<0.001	<0.001	0.00000 ± 0.00000 ^a^	0.00000 ± 0.00000 ^a^	0.00000 ± 0.00000 ^a^	0.04044 ± 0.00022 ^b^	0.00000 ± 0.00000 ^a^	0.00000 ± 0.00000 ^a^
L-Glycine (mg mL^−1^)	<0.001	<0.001	<0.001	0.00000 ± 0.00000 ^a^	0.00000 ± 0.00000 ^a^	0.07421 ± 0.00033 ^b^	0.00000 ± 0.00000 ^a^	0.00000 ± 0.00000 ^a^	0.00000 ± 0.00000 ^a^
L-Proline (mg mL^−1^)	<0.001	<0.001	<0.001	0.00000 ± 0.00000 ^a^	0.00000 ± 0.00000 ^a^	0.09723 ± 0.00172 ^b^	0.00000 ± 0.00000 ^a^	0.00000 ± 0.00000 ^a^	0.00000 ± 0.00000 ^a^
KI	<0.001	0.0617	<0.001	18.090 ± 0.750 ^ac^	14.716 ± 0.673 ^bd^	16.514 ± 1.639 ^ab^	19.827 ± 0.564 ^c^	15.801 ± 0.453 ^ab^	13.341 ± 0.516 ^d^
IBU	<0.001	<0.001	<0.001	18.000 ± 0.010 ^d^	26.708 ± 0.268 ^b^	12.617 ± 0.015 ^f^	23.433 ± 0.021 ^c^	36.560 ± 0.061 ^a^	13.292 ± 0.157 ^e^
Ethanol (%)	<0.001	<0.001	<0.001	6.473 ± 0.040 ^a^	4.561 ± 0.020 ^e^	8.112 ± 0.012 ^b^	7.230 ± 0.049 ^c^	6.805 ± 0.080 ^d^	6.418 ± 0.067 ^a^
Isoamyl alcohol (%)	0.3966	0.1564	0.0363	0.01567 ± 0.00058 ^a^	0.01400 ± 0.00000 ^a^	0.01533 ± 0.00058 ^a^	0.01533 ± 0.00058 ^a^	0.01500 ± 0.00100 ^a^	0.01533 ± 0.00058 ^a^
Isobutanol (%)	<0.001	0.2046	0.0414	0.18300 ± 0.00100 ^ab^	0.18267 ± 0.00058 ^ab^	0.18400 ± 0.00000 ^bc^	0.18533 ± 0.00058 ^c^	0.18200 ± 0.00000 ^a^	0.18200 ± 0.00000 ^a^

Different lowercase letters within the same row indicate significant differences among cereal × inclusion level combinations (Tukey’s honestly significant difference (HSD) test, *p* < 0.05). Cereal: wheat, rice, and sorghum; inclusion level: 20 and 40%. KI: Kolbach index; IBU: international bitterness units; TPC: total phenolic content; GAE: gallic acid equivalents; ORAC: oxygen radical absorbance capacity; TE: Trolox equivalents; ABTS: 2,2′-azinobis-(3-ethylbenzothiazoline-6-sulfonic acid). Values are expressed as mean ± standard deviation (*n* = 3), based on three independent brewing batches. The control was excluded from the factorial analysis.

**Table 7:** Sensory evaluation of Ale beer formulations produced with different unmalted cereal adjuncts.

Formulation	Overall Acceptability	Sweetness	Bitterness	Aroma	Body	Carbonation
Control	6.19 ± 1.58 ^a^	4.68 ± 1.83 ^ab^	5.19 ± 1.77 ^a^	5.90 ± 1.85 ^ab^	5.71 ± 1.73 ^a^	5.87 ± 1.80 ^abc^
T1	6.26 ± 1.81 ^a^	4.87 ± 2.02 ^ab^	5.90 ± 2.00 ^a^	5.56 ± 1.95 ^abc^	5.43 ± 1.84 ^ab^	6.42 ± 1.77 ^a^
T2	6.24 ± 1.76 ^a^	4.18 ± 1.72 ^b^	5.55 ± 1.92 ^a^	5.93 ± 1.71 ^a^	5.56 ± 1.93 ^ab^	5.73 ± 2.23 ^abc^
T3	5.82 ± 1.96 ^a^	4.54 ± 1.78 ^ab^	5.50 ± 1.84 ^a^	5.16 ± 1.68 ^abc^	5.30 ± 1.54 ^ab^	5.38 ± 1.56 ^c^
T4	5.99 ± 1.53 ^a^	4.71 ± 1.82 ^ab^	5.22 ± 2.01 ^a^	5.02 ± 1.97 ^c^	5.54 ± 1.56 ^ab^	5.60 ± 1.51 ^bc^
T5	4.95 ± 2.04 ^b^	4.14 ± 1.81 ^b^	5.41 ± 1.96 ^a^	5.12 ± 2.11 ^bc^	4.93 ± 1.68 ^b^	6.23 ± 1.94 ^ab^
T6	6.32 ± 1.53 ^a^	5.14 ± 1.47 ^a^	5.52 ± 1.77 ^a^	5.03 ± 1.84 ^c^	5.20 ± 1.47 ^ab^	5.64 ± 1.73 ^bc^

Values are expressed as mean ± standard deviation (*n* = 100 consumers). Different lowercase superscript letters within the same column indicate significant differences among beer formulations (Tukey’s honestly significant difference (HSD) test, *p* < 0.05).

**Table 8:** Formulations of Ale beers produced with different proportions of unmalted cereal adjuncts.

Formulation	Barley (%)	Rice (%)	Wheat (%)	Sorghum (%)
Control	100	0	0	0
T1	80	20	0	0
T2	60	40	0	0
T3	80	0	20	0
T4	60	0	40	0
T5	80	0	0	20
T6	60	0	0	40

## Data Availability

All the data related to this work are given here in the manuscript.

## Supplementary Materials

The following supporting information can be downloaded at: https://www.mdpi.com/article/10.3390/molecules31213935/s1, Table S1. Analysis of variance of sensory attributes of craft ale beers evaluated by consumers; Table S2. Calibration parameters and analytical validation parameters for volatile alcohols determined by GC-FID; Table S3. UPLC–ESI–MS operating parameters used for the determination of free amino acids.

## References

[1] Matraxia M., Alfonzo A., Prestianni R., Francesca N., Gaglio R., Todaro A., Alfeo V., Perretti G., Columba P., Settanni L. (2021). Non-conventional yeasts from fermented honey by-products: Focus on *Hanseniaspora uvarum* strains for craft beer production. Food Microbiol..

[2] Mosher M., Trantham K. (2021). Brewing Science: A Multidisciplinary Approach.

[3] Bertuzzi T., Mulazzi A., Rastelli S., Donadini G., Rossi F., Spigno G. (2020). Targeted healthy compounds in small and large-scale brewed beers. Food Chem..

[4] Tozetto L.M., Nascimento R.F.D., De Oliveira M.H., Van Beik J., Canteri M.H.G. (2019). Production and physicochemical characterization of craft beer with ginger (*Zingiber officinale*). Food Sci. Technol..

[5] Villacreces S., Blanco C.A., Caballero I. (2022). Developments and characteristics of craft beer production processes. Food Biosci..

[6] Garavaglia C. (2025). Definition, history, and market of craft beer. Craft Beer.

[7] Pereira M.J., Santos D., Cruz A., Jesus Â., Martins J.P., Moreira F., Santos M., Pinho C., Oliveira A.I. (2025). Exploring Alternative Potentialities of Portuguese and Spanish Craft Beers: Antioxidant and Photoprotective Activities. Beverages.

[8] Market Data Forecast (2026). Craft Beer Market Size, Share, Trends & Analysis, 2034. https://www.marketdataforecast.com/market-reports/craft-beer-market.

[9] Bokulich N.A., Bamforth C.W. (2013). The Microbiology of Malting and Brewing. Microbiol. Mol. Biol. Rev..

[10] Ualema N.J.M., dos Santos L.N., Bogusz S., Ferreira N.R. (2024). From Conventional to Craft Beer: Perception, Source, and Production of Beer Color—A Systematic Review and Bibliometric Analysis. Foods.

[11] Liguori L., De Francesco G., Orilio P., Perretti G., Albanese D. (2020). Influence of malt composition on the quality of a top fermented beer. J. Food Sci. Technol..

[12] Castro L.F., Affonso A.D., Lehman R.M. (2021). Impact of Specialty Malts on Wort and Beer Characteristics. Fermentation.

[13] Cadenas R., Caballero I., Nimubona D., Blanco C.A. (2021). Brewing with Starchy Adjuncts: Its Influence on the Sensory and Nutritional Properties of Beer. Foods.

[14] Dabija A., Ciocan M.E., Chetrariu A., Codină G.G. (2021). Maize and Sorghum as Raw Materials for Brewing, a Review. Appl. Sci..

[15] Poreda A., Czarnik A., Zdaniewicz M., Jakubowski M., Antkiewicz P. (2014). Corn grist adjunct—Application and influence on the brewing process and beer quality. J. Inst. Brew..

[16] Rani H., Bhardwaj R.D. (2021). Quality attributes for barley malt: “The backbone of beer”. J. Food Sci..

[17] Bogdan P., Kordialik-Bogacka E. (2017). Alternatives to malt in brewing. Trends Food Sci. Technol..

[18] Nardini M. (2023). An Overview of Bioactive Phenolic Molecules and Antioxidant Properties of Beer: Emerging Trends. Molecules.

[19] Habschied K., Krstanović V., Mastanjević K. (2022). Beer Quality Evaluation—A Sensory Aspect. Beverages.

[20] Addo-Preko E., Amissah J.G.N., Adjei M.Y.B. (2023). The relevance of the number of categories in the hedonic scale to the Ghanaian consumer in acceptance testing. Front. Food Sci. Technol..

[21] Wang Y., Zhao X., Liu S., Zhu J., Yin Y., Yang Z. (2025). Impact of Brown Rice as Adjunct on Beer Brewing. Foods.

[22] De Flaviis R., Santarelli V., Sacchetti G. (2022). Tracking Wheat Variety and Origin by the Shape Analysis of the Volatiles Fingerprint of Wheat Kernels and Wheat Beers. Appl. Sci..

[23] Schnitzenbaumer B., Arendt E.K. (2014). Brewing with up to 40% Unmalted Oats (*Avena sativa*) and Sorghum (*Sorghum bicolor*): A Review. J. Inst. Brew..

[24] Yorke J., Cook D., Ford R. (2021). Brewing with Unmalted Cereal Adjuncts: Sensory and Analytical Impacts on Beer Quality. Beverages.

[25] Cela N., Galgano F., Perretti G., Di Cairano M., Tolve R., Condelli N. (2022). Assessment of Brewing Attitude of Unmalted Cereals and Pseudocereals for Gluten Free Beer Production. Food Chem..

[26] Baillière J., Laureys D., Vermeir P., Van Opstaele F., De Rouck G., De Cooman L., Vanderputten D., De Clippeleer J. (2022). 10 Unmalted Alternative Cereals and Pseudocereals: A Comparative Analysis of Their Characteristics Relevant to the Brewing Process. J. Cereal Sci..

[27] Paszkot J., Gasiński A., Kawa-Rygielska J. (2023). Evaluation of volatile compound profiles and sensory properties of dark and pale beers fermented by different strains of brewing yeast. Sci. Rep..

[28] Pires E., Brányik T. (2015). Biochemistry of Beer Fermentation.

[29] Evans E. (2005). Brewing: Science and Practice, by Briggs, D.E.; Boulton, C.A.; Brookes, P.A.; Stevens, R.; Woodhead Publishing: Cambridge, UK; CRC Press: Boca Raton, FL, USA, 2004; 881p. J. Sci. Food Agric..

[30] Sayre-Chavez B., Bettenhausen H., Windes S., Aron P., Cistué L., Fisk S., Helgerson L., Heuberger A.L., Tynan S., Hayes P. (2022). Genetic basis of barley contributions to beer flavor. J. Cereal Sci..

[31] Villicaña M.T.O., Saldivar S.O.S. (2004). Production of Lager Beer from Sorghum Malt and Waxy Grits. J. Am. Soc. Brew. Chem..

[32] Bellut K., Michel M., Zarnkow M., Hutzler M., Jacob F., Lynch K.M., Arendt E.K. (2019). On the suitability of alternative cereals, pseudocereals and pulses in the production of alcohol-reduced beers by non-conventional yeasts. Eur. Food Res. Technol..

[33] Gupta M., Abu-Ghannam N., Gallaghar E. (2010). Barley for brewing: Characteristic changes during malting, brewing and applications of its by-products. Compr. Rev. Food Sci. Food Saf..

[34] Kunze W. (2014). Technology Brewing and Malting.

[35] Bamforth C.W. (2023). Beer: Tap into the Art and Science of Brewing.

[36] Ceccaroni D., Sileoni V., Marconi O., De Francesco G., Lee E.G., Perretti G. (2018). Specialty rice malt optimization and improvement of rice malt beer aspect and aroma. LWT.

[37] Steiner E., Gastl M., Becker T. (2011). Protein changes during malting and brewing with focus on haze and foam formation: A review. Eur. Food Res. Technol..

[38] Taylor J.R., Duodu K.G. (2017). Sorghum and Millets: Grain-Quality Characteristics and Management of Quality Requirements. Cereal Grains.

[39] Duodu K., Taylor J., Belton P., Hamaker B. (2003). Factors affecting sorghum protein digestibility. J. Cereal Sci..

[40] Hazelwood L.A., Daran J.M., van Maris A.J.A., Pronk J.T., Dickinson J.R. (2008). The Ehrlich pathway for fusel alcohol production. Appl. Environ. Microbiol..

[41] Saerens S.M.G., Delvaux F.R., Verstrepen K.J., Thevelein J.M. (2010). Production and biological function of volatile esters in *Saccharomyces cerevisiae*. Microb. Biotechnol..

[42] Verbelen P.J., De Schutter D.P., Delvaux F., Verstrepen K.J., Delvaux F.R. (2006). Immobilized yeast cell systems for continuous fermentation applications. Biotechnol. Lett..

[43] Dufour J.P., Mélotte L., Srebrnik S. (1992). Sorghum Malts for the Production of a Lager Beer. J. Am. Soc. Brew. Chem..

[44] Taylor J.R.N., Dlamini B.C., Kruger J. (2013). 125th Anniversary Review: The science of the tropical cereals sorghum, maize and rice in relation to lager beer brewing. J. Inst. Brew..

[45] Titze J., Ilberg V., Jacob F., Parlar H. (2005). A comparison of the EBC 9.8 method with a common applied HPLC analysis for determining bitterness in beer. BrewingScience.

[46] Klimczak K., Cioch-Skoneczny M. (2023). Changes in beer bitterness level during the beer production process. Eur. Food Res. Technol..

[47] Awika J.M., Rooney L.W. (2004). Sorghum phytochemicals and their potential impact on human health. Phytochemistry.

[48] Lekkas C., Stewart G.G., Hill A.E., Taidi B., Hodgson J.A. (2005). The Importance of Free Amino Nitrogen in Wort and Beer. MBAA Tech. Q..

[49] Walker G.M. (2011). *Pichia anomala*: Cell physiology and biotechnology relative to other yeasts. Antonie Van Leeuwenhoek.

[50] Bell S., Henschke P.A. (2005). Implications of nitrogen nutrition for grapes, fermentation and wine. Aust. J. Grape Wine Res..

[51] Kunz T., Müller C., Mato-Gonzales D., Methner F. (2012). The influence of unmalted barley on the oxidative stability of wort and beer. J. Inst. Brew..

[52] Beta T., Rooney L., Marovatsanga L., Taylor J. (2000). Effect of Chemical Treatments on Polyphenols and Malt Quality in Sorghum. J. Cereal Sci..

[53] Everette J.D., Bryant Q.M., Green A.M., Abbey Y.A., Wangila G.W., Walker R.B. (2010). Thorough Study of Reactivity of Various Compound Classes toward the Folin–Ciocalteu Reagent. J. Agric. Food Chem..

[54] Huang D., Ou B., Prior R.L. (2005). The Chemistry behind Antioxidant Capacity Assays. J. Agric. Food Chem..

[55] Nardini M., Foddai M.S. (2020). Phenolics Profile and Antioxidant Activity of Special Beers. Molecules.

[56] Carvalho D.O., Gonçalves L.M., Guido L.F. (2016). Overall Antioxidant Properties of Malt and How They Are Influenced by the Individual Constituents of Barley and the Malting Process. Compr. Rev. Food Sci. Food Saf..

[57] Prior R.L., Wu X., Schaich K. (2005). Standardized methods for the determination of antioxidant capacity and phenolics in foods and dietary supplements. J. Agric. Food Chem..

[58] Xu J., Wang W., Zhao Y. (2021). Phenolic compounds in whole grain sorghum and their health benefits. Foods.

[59] Adom K.K., Liu R.H. (2002). Antioxidant Activity of Grains. J. Agric. Food Chem..

[60] Zhao H., Chen W., Lu J., Zhao M. (2009). Phenolic profiles and antioxidant activities of commercial beers. Food Chem..

[61] Re R., Pellegrini N., Proteggente A., Pannala A., Yang M., Rice-Evans C. (1999). Antioxidant activity applying an improved ABTS radical cation decolorization assay. Free Radic. Biol. Med..

[62] Awika J.M., Rooney L.W., Wu X., Prior R.L., Cisneros-Zevallos L. (2003). Screening Methods To Measure Antioxidant Activity of Sorghum (*Sorghum bicolor*) and Sorghum Products. J. Agric. Food Chem..

[63] Yu L., Haley S., Perret J., Harris M., Wilson J., Qian M. (2002). Free Radical Scavenging Properties of Wheat Extracts. J. Agric. Food Chem..

[64] Gorjanović S., Pastor F.T., Vasić R., Novaković M., Simonović M., Milić S., Sužnjević D. (2013). Electrochemical versus Spectrophotometric Assessment of Antioxidant Activity of Hop (*Humulus lupulus* L.) Products and Individual Compounds. J. Agric. Food Chem..

[65] Zhao X., Yin Y., Fang W., Yang Z. (2023). Potential of germinated brown rice in beer brewing. J. Cereal Sci..

[66] Gasiński A., Kawa-Rygielska J., Spychaj R., Opiela E., Sowiński J. (2023). Production of gluten-free beer brewing from sorghum malts mashed without external enzyme preparations. J. Cereal Sci..

[67] Myncke E., Vandoorne S., Huys J., Smout P., Laureys D., Vermeir P., Schlich J., Van Opstaele F., Vanderputten D., De Clippeleer J. (2026). Wort optimisation for non-alcoholic beer production with maltose-negative yeasts: Incorporation of malted, flaked, and unmalted adjuncts. Food Chem..

[68] Kovács T., Papp D.A., Varga M., Szekeres A., Palágyi A., Vágvölgyi C., Papp T., Krisch J., Takó M. (2024). Influence of enzyme treatment approach on the phenolics content and antioxidant potential of sorghum grain samples. LWT.

[69] Paes L.T., D’Almeida C.T.S., do Carmo M.A.V., da Silva Cruz L., de Souza A.B., Viana L.M., Maltarollo V.G., Martino H.S.D., Lima G.D.A., Ferreira M.S.L. (2024). Phenolic-rich extracts from toasted white and tannin sorghum flours have distinct profiles influencing their antioxidant, antiproliferative, anti-adhesive, anti-invasive, and antimalarial activities. Food Res. Int..

[70] Habschied K., Košir I.J., Krstanović V., Kumrić G., Mastanjević K. (2021). Beer Polyphenols—Bitterness, Astringency, and Off-Flavors. Beverages.

[71] McMurrough I., Madigan D., Kelly R.J., Smyth M.R. (1996). The Role of Flavanoid Polyphenols in Beer Stability. J. Am. Soc. Brew. Chem..

[72] Oladokun O., Tarrega A., James S., Cowley T., Dehrmann F., Smart K., Cook D., Hort J. (2016). Modification of perceived beer bitterness intensity, character and temporal profile by hop aroma extract. Food Res. Int..

[73] Boulton C., Quain D. (2001). Brewing Yeast and Fermentation.

[74] Šimečková N., Gregor T., Hřivna L., Kouřilová V., Dufková R., Simonová J., Burešová I. (2024). Quality of beer with added pseudocereals. Kvas. Prum..

[75] Piazzon A., Forte M., Nardini M. (2010). Characterization of phenolics content and antioxidant activity of different beer types. J. Agric. Food Chem..

[76] Liu J., Chu B., Yang X., Jin Y. (2021). Relationship between the index of protein modification (Kolbach index) and degradation of macromolecules in wheat malt. J. Food Sci..

[77] AOAC (2005). Official Methods of Analysis.

[78] Latimer G.W., AOAC International (2023). AOAC Official Method 985.29: Total Dietary Fiber in Foods. Official Methods of Analysis of AOAC International.

[79] Ponce-Ramirez J.A., Horna-Menacho N., Rodríguez G., Barco-Jara M.S., Villanueva E., Hurtado-Soria B.Z., Ortecho-Llanos R., De Fátima Arevalo-Oliva M., Tapia-Blácido D.R., Aguirre E. (2026). Valorization of Artichoke Bract Flour as Reinforcement in Biodegradable Cassava Starch Trays: Physicochemical, Structural, and Thermo-Mechanical Properties. Carbohydr. Polym. Technol. Appl..

[80] ASBC (2011). Beer Bitterness.

[81] Mejia-Llontop C.I., Tirado-Rodríguez C.E., Acosta-Baca A., Aguayo-Flores M., Ascate-Pasos M., Ayala-Jara C., Rodriguez G., Villanueva E., Aguirre E. (2025). Impact of Hop Residue Reuse on the Chemical and Sensory Properties of Craft Beer. Resources.

[82] European Brewery Convention (2007). Analytica—EBC Methods of Analysis.

[83] Johnson B.A., Johansson C.-G. (1999). Determination of the Total Soluble Nitrogen Content of Malt and Beer by the Dumas Combustion Method: Collaborative Trial. J. Inst. Brew..

[84] Saura-Calixto F., Serrano J., Goñi I. (2007). Intake and bioaccessibility of total polyphenols in a whole diet. Food Chem..

[85] Prior R.L., Hoang H., Gu L., Wu X., Bacchiocca M., Howard L., Hampsch-Woodill M., Huang D., Ou B., Jacob R. (2003). Assays for hydrophilic and lipophilic antioxidant capacity (oxygen radical absorbance capacity (ORACFL)) of plasma and other biological and food samples. J. Agric. Food Chem..

