# Supplementary material for: Unmalted Rice, Wheat, and Sorghum Modulate Antioxidant Capacity, Nitrogen Metabolism, Alcohol Profile, and Sensory Quality of Craft Ale Beer
Source: Molecules. 2026 Nov 1;31(21):3935. doi: 10.3390/molecules31213935 (PMC13764775; doi:10.3390/molecules31213935)
Supplement: Supplementary file 1 [file molecules-31-03935-s001.zip › molecules-4656837-supplementary.pdf]

Supplementary Materials

Table S1. Analysis of variance of sensory attributes of craft ale beers evaluated by consumers.

Table S2. Calibration parameters and analytical validation parameters for volatile alcohols determined by GC-FID.

Table S3. UPLC–ESI–MS operating parameters used for the determination of free amino acids.

Table S1. Analysis of variance of sensory attributes of craft ale beers evaluated by consumers.

Sensory attribute	Source	df	Adjusted SS	Adjusted MS	F-value	*p*-value
**Overall acceptability**	Formulations	6	138.20	23.029	7.78	**<0.001**
	Consumer	99	372.30	3.760	1.27	0.051
	Error	594	1758.90	2.961	—	—
	Total	699	2269.40	—	—	—
**Sweetness**	Formulations	6	77.46	12.910	4.07	**0.001**
	Consumer	99	324.65	3.279	1.03	0.400
	Error	594	1884.50	3.173	—	—
	Total	699	2286.61	—	—	—
**Bitterness**	Formulations	6	33.51	5.585	1.59	0.148
	Consumer	99	410.86	4.150	1.18	0.126
	Error	594	2086.45	3.513	—	—
	Total	699	2530.81	—	—	—
**Aroma**	Formulations	6	96.61	16.101	4.59	**<0.001**
	Consumer	99	360.89	3.645	1.04	0.388
	Error	594	2084.65	3.510	—	—
	Total	699	2542.15	—	—	—
**Body**	Formulations	6	41.273	6.879	2.38	0.028
	Consumer	99	250.945	2.535	0.88	0.79
	Error	594	1717.761	2.892		
	Total	699	2009.98			
**Carbonation**	Formulations	6	80.228	13.371	4.07	<0.001
	Consumer	99	307.504	3.106	0.95	0.626
	Error	594	1950.349	3.283	—	—
	Total	699	2338.081	—	—	—

df, degrees of freedom; SS, sum of squares; MS, mean square. Bold p-values indicate statistically significant effects at $p < 0.05$.

Table S2. Calibration parameters and analytical validation parameters for volatile alcohols determined by GC-FID.

Compound	Slope (*m*)	Intercept (*b*)	R^2	$S_y/_x$	LOD (%, *v v*$^{-1}$)	LOQ (%, *v v*$^{-1}$)	ME (%)	Recovery (%)
**Ethanol**	133,030.934	8.549	0.999	9.712	0.000241	0.000730	99.68	0.966
**Isobutanol**	263,671.685	20.224	0.999	25.448	0.000319	0.000965	100.34	0.933
**Isoamyl alcohol**	295,668.591	15.819	0.999	19.294	0.000215	0.000653	102.23	0.954

R^2, coefficient of determination; $S_y/_x$, residual standard deviation of the regression; LOD, estimated limit of detection; LOQ, estimated limit of quantification; ME, matrix effect.

Table S3. UPLC–ESI–MS operating parameters used for the determination of free amino acids.

Amino acid	Precursor ion (m/z)	Fragmentor voltage (V)	Calibration equation	R^2	Recovery (%)	LOD (μg/mL)	LOQ (μg/mL)	ME (%)
L-Cysteine	241	105	$y = 1.17521 \times 10^4x + 6.73188 \times 10^5$	0.997	95.34	17.315	52.471	104.0
L-Tyrosine	182.1	95	$y = 9.24705 \times 10^4x + 2.16624 \times 10^6$	0.998	100.12	8.439	25.572	101.0
L-Arginine	175.1	105	$y = 5.51412 \times 10^4x + 2.23370 \times 10^6$	0.998	113.25	11.073	33.555	95.0
L-Aspartic acid	134.1	75	$y = 2.05128 \times 10^3x + 1.44613 \times 10^5$	0.998	99.59	8.245	24.985	102.0
L-Isoleucine	132.1	85	$y = 2.63254 \times 10^5x + 1.28210 \times 10^7$	0.997	106.08	10.385	31.468	96.0
L-Leucine	132.1	85	$y = 3.08721 \times 10^5x + 1.26988 \times 10^7$	0.997	90.74	9.353	28.343	105.0
L-Threonine	120	75	$y = 7.02670 \times 10^3x + 6.81143 \times 10^5$	0.998	102.15	7.576	22.957	103.0
L-Valine	118	75	$y = 2.11940 \times 10^5x - 5.08190 \times 10^5$	0.999	92.53	4.019	12.180	101.0
L-Proline	116.1	85	$y = 2.18872 \times 10^5x - 4.73961 \times 10^6$	0.997	91.81	30.491	92.396	97.0
L-Serine	106.1	65	$y = 5.43983 \times 10^3x + 3.56632 \times 10^5$	0.999	99.27	3.304	10.013	98.0
L-Alanine	90.1	90	$y = 3.20392 \times 10^3x + 4.70968 \times 10^5$	0.997	90.79	7.021	21.276	102.0
L-Glycine	76	120	$y = 2.38054 \times 10^3x + 1.07571 \times 10^5$	0.998	117.06	5.121	15.519	100.0

Note: *m*/*z*, mass-to-charge ratio; ESI (+), positive electrospray ionization mode, used for all amino acids analyzed; LOD, limit of detection; LOQ, limit of quantification. Calibration curves were constructed using five concentration levels (10, 25, 100, 250, and 1000 pmol mL^{-1}), with four replicate injections at each level ($n = 4$). The calibration equation was expressed as $y = Sx + b$, where y is the peak area, x is the amino acid concentration (pmol mL^{-1}), S is the slope, and bb is the y-intercept. LOD and LOQ were calculated in molar concentration according to $LOD = 3.3\sigma/S$ and $LOQ = 10\sigma/S$, respectively, where σ is the standard deviation of the peak area at the lowest calibration level (10 pmol mL^{-1}, $n = 4$) and S is the slope of the corresponding calibration curve. The resulting values were subsequently converted to mass concentration (μg mL^{-1}) using the molecular weight of each amino acid or, for L-lysine·HCl, the molecular weight of the hydrochloride salt. L-cysteine was quantified as its oxidized dimer, L-cystine ($[M+H]^+$, *m*/*z* 241.0), and the molecular weight of cystine was used for the corresponding conversion. ME, matrix effect.
